# Label-free spatially maintained measurements of metabolic phenotypes in cells

**DOI:** 10.3389/fbioe.2023.1293268

**Published:** 2023-11-28

**Authors:** Linghao Hu, Nianchao Wang, Joshua D. Bryant, Lin Liu, Linglin Xie, A. Phillip West, Alex J. Walsh

**Affiliations:** ^1^ Department of Biomedical Engineering, Texas A&M University, College Station, TX, United States; ^2^ Microbial Pathogenesis and Immunology, Health Science Center, Texas A&M University, College Station, TX, United States; ^3^ Department of Nutrition, Texas A&M University, College Station, TX, United States; ^4^ Department of Integrative Physiology, Baylor College of Medicine, Houston, TX, United States

**Keywords:** autofluorescence, fluorescence lifetime, machine learning, metabolism, cancer

## Abstract

Metabolic reprogramming at a cellular level contributes to many diseases including cancer, yet few assays are capable of measuring metabolic pathway usage by individual cells within living samples. Here, autofluorescence lifetime imaging is combined with single-cell segmentation and machine-learning models to predict the metabolic pathway usage of cancer cells. The metabolic activities of MCF7 breast cancer cells and HepG2 liver cancer cells were controlled by growing the cells in culture media with specific substrates and metabolic inhibitors. Fluorescence lifetime images of two endogenous metabolic coenzymes, reduced nicotinamide adenine dinucleotide (NADH) and oxidized flavin adenine dinucleotide (FAD), were acquired by a multi-photon fluorescence lifetime microscope and analyzed at the cellular level. Quantitative changes of NADH and FAD lifetime components were observed for cells using glycolysis, oxidative phosphorylation, and glutaminolysis. Conventional machine learning models trained with the autofluorescence features classified cells as dependent on glycolytic or oxidative metabolism with 90%–92% accuracy. Furthermore, adapting convolutional neural networks to predict cancer cell metabolic perturbations from the autofluorescence lifetime images provided improved performance, 95% accuracy, over traditional models trained via extracted features. Additionally, the model trained with the lifetime features of cancer cells could be transferred to autofluorescence lifetime images of T cells, with a prediction that 80% of activated T cells were glycolytic, and 97% of quiescent T cells were oxidative. In summary, autofluorescence lifetime imaging combined with machine learning models can detect metabolic perturbations between glycolysis and oxidative metabolism of living samples at a cellular level, providing a label-free technology to study cellular metabolism and metabolic heterogeneity.

## 1 Introduction

Despite the relevance of cellular metabolism to normal and pathological physiology, a technological gap exists for methods that measure metabolism in living samples with single-cell resolution. Cellular metabolism is linked with cellular function for many cell types. For example, cancer is often characterized by a dependence on aerobic glycolysis, and the function of many immune cells, including pro-inflammatory macrophages and activated T cells, is dependent on glycolysis (Warburg, 1956; Leone and Powell, 2020). Single-cell RNA analysis, a technique that allows single-cell measurements but requires tissue dissociation and fixation, has revealed high metabolic heterogeneity exists within tissues and can affect patient outcomes; for example, metabolic heterogeneity is correlated with increased cancer metastasis (Davis et al., 2020; Hartmann et al., 2021). Measurements of metabolism on live cells, such as with oxygen consumption assays which can be performed on live cells but lack single-cell resolution, demonstrate that cellular metabolic pathway use is dynamic and responsive to microenvironments, substrate availability, and chemical signaling (Dar et al., 2017; Masoud et al., 2020; Nile et al., 2021). Therefore, full characterization of cellular metabolism and metabolic heterogeneity within tissues requires a technique that provides metabolic information, can be performed on live cells, allows repeated measurements to capture changes or dynamics, maintains the spatial positioning of the cells, and has single-cell or better resolution. Such a technology will be vital for advancing mechanistic and therapeutic research within the myriad of metabolic-relevant pathologies, including cancer development and metastasis, diabetes, and neurodegeneration. To address this technological gap, here, the combined use of autofluorescence lifetime imaging, single-cell segmentation, and machine learning (ML) models is evaluated to predict the metabolic pathway use of single cells.

Currently, metabolic measurement technologies are limited in spatial and temporal resolution. The oxygen consumption rate (OCR) and the extracellular acidification rate (ECAR) of cell populations can be used to evaluate mitochondrial respiration and glycolysis, respectively (Zhang et al., 2012; Van den Bossche et al., 2015). However, these measurements are recorded for cell populations. Likewise, biochemical analyses of metabolic enzymes, including Western Blot analysis, mass spectroscopy, mRNA analysis, and immunohistochemistry, require cell or tissue fixation and generally lack single-cell resolution (Ramm Sander et al., 2013). Positron Emission tomography (PET) detects radioactive substances, such as 2-deoxy-2-[18F] fluoro-D-glucose (FDG) to visualize glucose uptake of tumors which is higher than surrounding tissue due to enhanced glycolysis (Challapalli and Aboagye, 2016). FDG-PET is used clinically, and additional contrast agents are in development to detect choline metabolism, glutamine transport, and fatty acid metabolism (Steven et al., 1996; Hara et al., 1997; Lieberman et al., 2011). However, the spatial resolution of PET is fundamentally limited by the millimeter distances that positrons can travel prior to annihilation events (Moses, 2011). Signaling pathways can be labeled with genetically-encoded fluorescence proteins to offer metabolic information, however, the dependence on exogenous fluorescent labels requires cellular manipulations and brings confounding factors like cell destruction, dye concentration, and label distribution errors (Kauffman et al., 2016; Little et al., 2020). Raman spectra and microscopy can detect chemical bond signatures distinct to lipids, fatty acids, phospholipids, amino acids, and proteins at a single-cell level in a label-free manner (Okada et al., 2012; Yue and Cheng, 2016). Raman microscopy can also be used to measure carbon metabolism in live cells, but this typically relies on labeled glucose (Li and Cheng, 2014; Hu et al., 2015; Xu et al., 2017). Therefore, a robust technology capable of live-cell metabolic measurements remains elusive, yet potentially impactful due to a large number of diseases and pathologies characterized by metabolic dysfunction and heterogeneity.

Autofluorescence imaging of two key endogenous metabolic co-enzymes reduced nicotinamide adenine dinucleotide (NADH) and oxidized flavin adenine dinucleotide (FAD) offers functional metrics for detecting metabolic variations (Chance et al., 1979; Georgakoudi and Quinn, 2012). NADH and FAD are used in metabolic pathways including glycolysis, oxidative phosphorylation (OXPHOS), and glutaminolysis. Glycolysis breaks down glucose into pyruvate and reduces NAD + to NADH in the cytosol. This can be used to reduce mitochondrial NAD^+^ to NADH through the malate-aspartate shuttle. This, alongside NADH produced from the further oxidation of pyruvate inside the mitochondrial TCA cycle, is used to generate ATP via the electron transport chain. During glutaminolysis, NAD^+^ also assists glutamate dehydrogenase (GDH) to convert glutamate to α-ketoglutarate by reducing to NADH. The fluorescence intensity ratio of NADH and FAD is defined as the optical redox ratio and has been widely used as a marker of the redox state in cells and tissues (Chance et al., 1979; Skala et al., 2007). The spectral properties of NADH and its phosphorylated form, NADPH are identical, thus NAD(P)H is used to represent their combined fluorescence signal detected from cells. Similarly, the fluorescence of different flavin species such as flavin mononucleotide (FMN) and FAD are challenging to separate due to the substantial spectral overlap of their fluorescence properties (Gregor et al., 2018). FAD is the predominate intracellular fluorescent flavin molecule, with FMN contributing a small fraction, around 5% as previously estimated, of the flavin autofluorescence signal (Huhner et al., 2015; Kalinina et al., 2021). Due to the primary contribution of FAD to flavin autofluorescence signals and for consistency with prior optical metabolic imaging literature, the flavin autofluorescence is hereafter referred to as FAD, and potential errors in data interpretation due to this convention are discussed where appropriate (Georgakoudi and Quinn, 2012; Kolenc and Quinn, 2019).

The fluorescence lifetime is the time a fluorophore remains in the excited state before releasing a fluorescent photon and resolves information on chemical structures and the surrounding microenvironments of NAD(P)H and FAD (Nakashima et al., 1980; Jr et al., 1992; Lakowicz, 2006). Both NAD(P)H and FAD can exist in two conformations, protein-bound or free within cells, each of which has a different fluorescence lifetime (Nakashima et al., 1980; Jr et al., 1992). Fluorescence lifetime imaging (FLIM) resolves the fraction of free and protein-bound coenzymes as well as the corresponding short- and long-lifetime components (Becker, 2012; Georgakoudi and Quinn, 2012). The fluorescence intensity and lifetime of NAD(P)H and FAD are sensitive to metabolic changes in precancerous tissues, disease development, drug treatment responses of cancer cells, differentiation of stem cells, and macrophage phenotype (Bird et al., 2005; Skala et al., 2007; Ostrander et al., 2010; Konig et al., 2011; Stringari et al., 2012b; Quinn et al., 2013; Walsh et al., 2013; Colin et al., 2014; Varone et al., 2014; Palmer et al., 2015; Alfonso-Garcia et al., 2016; Szulczewski et al., 2016; Fayad et al., 2018; Borowczyk et al., 2020; Heaster et al., 2020). Moreover, cell segmentation from the NAD(P)H intensity images provides single-cell and subcellular information and quantifies cellular heterogeneity (Walsh et al., 2021; Cardona and Walsh, 2022).

Although autofluorescence imaging of NAD(P)H and FAD often detects metabolic perturbations between samples and has been applied to identify various metabolic shifts in cells, the lifetime metrics lack specificity for direct interpretation of metabolic pathway usage. In this paper, alternations in NAD(P)H and FAD fluorescence lifetime features are defined for metabolic pathway perturbations of cancer cells using both chemical inhibition and substrate manipulation of glycolysis, oxidative phosphorylation, and glutaminolysis. The autofluorescence lifetime data is combined with one-dimension (1D) conventional machine learning (ML) algorithms and two-dimension (2D) convolutional neural networks (CNN) to predict glycolytic or oxidative phenotypes of cells. The 2D CNNs can learn spatial information from intensity and lifetime images and exhibit better performance in discriminating metabolic activities for larger datasets. The feature-based 1D ML models and 2D CNNs were then evaluated across different cell types to test model robustness and transference. Notably, cancer cells were used as a model system due to their high metabolic activity and ease of manipulation using substrates and inhibitors, and show that the results obtained from breast cancer cells can be extrapolated to liver cancer cells and non-cancerous T cells. These findings suggest that machine learning algorithms applied to autofluorescence lifetime images offer a reliable method for assessing glycolytic and oxidative phenotypes with high classification accuracy. Thus, these results and metabolism-prediction models will promote the application of autofluorescence lifetime imaging as a label-free, non-contact assay of cellular metabolism to provide important insights into biological and medical fields that study metabolism.

## 2 Materials and methods

### 2.1 Cell culture and preparation

MCF7 breast cancer cells were cultured in high glucose Dulbecco’s Modified Eagle’s Medium (DMEM), supplemented with 1% antibiotic-antimycotic, and 10% fetal bovine serum (FBS). For fluorescence lifetime imaging, cells were seeded at a density of 2 × 10^5^ per 35 mm glass-bottom imaging dish 48 h before imaging. Each dish was refreshed with the culture media 30 min before imaging to ensure constant consistent nutrient concentrations while imaging. To focus on the effects of glycolysis, OXPHOS, pyruvate concentration, and glutaminolysis on NAD(P)H and FAD fluorescence lifetime, specific inhibitors and substrates were applied to isolate the influence of each metabolic pathway. The metabolic perturbation details can be found in the Supplementary Material Appendix. HepG2 hepatoma cells were cultured in low glucose (5.6 mM) DMEM supplemented with 1% penicillin-streptomycin, and 10% FBS. The cells were seeded at a density of 2 × 10^5^ per 35 mm glass-bottom imaging dish and fasted for 24 h. Two metabolic groups were prepared by treating the cells with either 30 mM glucose or 0.4 mM palmitate (PA) respectively 12 h before imaging. The NAD(P)H and FAD fluorescence lifetime images from the activated and quiescent T cells were provided by AJ Walsh and MC Skala (Walsh et al., 2021).

### 2.2 Fluorescence lifetime imaging and analysis

The NAD(P)H and FAD fluorescence lifetime images were obtained using a custom-built multi-photon microscope (Marianas, 3i) coupled with a 40× water immersion objective (1.1 NA) and a tunable (680nm–1080 nm) Ti: sapphire femtosecond laser (COHERENT, Chameleon Ultra II). All cell imaging dishes were placed in a stage-top incubator (okolab) to maintain the environment of the cells at 37 °C, 5% CO_2_, and 85% relative humidity while imaging. NAD(P)H fluorescence was excited at 750 nm with a laser power of 6.8 mW–7.4 mW at the specimen plane. FAD fluorescence was excited at 890 nm with a laser power of 6.7 mW–7.5 mW at the specimen plane. To isolate NAD(P)H and FAD fluorescence emission, a 447/60 nm bandpass filter and a 550/88 nm bandpass filter, respectively, were placed before each detector. The bandpass filters were chosen based on the emission spectra of NAD(P)H and FAD to separate the two fluorophores and maximize the bandwidth to capture sufficient signal (Becker, 2021). Fluorescence lifetime images of NAD(P)H and FAD were obtained sequentially by photomultiplier tube (PMT) detectors (HAMAMATSU) attached to a time-correlated single-photon counting (TCSPC) electronics module (SPC-150N, Becker & Hickl). Each fluorescence lifetime image (256 × 256 pixels, 270 × 270 µm) was acquired with a pixel dwell time of 50 µs and 5 frame repeats for a collection time of 60 s. Both NAD(P)H and FAD fluorescence lifetime images were captured in at least five randomly selected positions for each dish, and three technical replicates were performed to ensure the reliability of the results. The second harmonic generated signal of urea crystals was excited at 900 nm and measured with the NAD(P)H channel for the instrument response function (IRF). Fluorescence lifetime measurements of the system were validated with a YG fluorescent bead, which had a measured lifetime of 2.1 ns, consistent with previously published values (Bird et al., 2005).

### 2.3 Cell-based image analysis

Fluorescence lifetime decays were analyzed by SPCImage (Becker & Hickl). Different thresholds were used to exclude pixels with low fluorescence intensity in NAD(P)H (minimum threshold of peak = 20 photons) and FAD (minimum threshold of peak = 3 photons) fluorescence lifetime images. The average number of photons at each cytoplasm pixel is around 1,000 with a peak photon above 100 in cancer cells with a spatial binning of 9 pixels. The average χ^2^ for fitting each image is around 1.07 for NAD(P)H images, and around 0.79 for FAD images. As the nucleus regions typically exhibit lower peak pixel values (50 for NAD(P)H, 20 for FAD), a low peak number threshold was used to ensure that all pixels in the cellular regions were included in the analysis. The lifetime value of each cell was then calculated based on the segmented cytoplasm regions, which had decay peaks with >100 photons. A binning of nine surrounding pixels was used, and the decay curve of each pixel was deconvoluted from the measured IRF of urea crystals and fitted to a two-component exponential model, $I\left(t\right)=\alpha_{1}e^{-t/\tau_{1}}+\alpha_{2}e^{-t/\tau_{2}}+C$ , where *I*(*t*) represents the fluorescence intensity as a function of time *t*, *α*_*1*_, *α*_*2*_ are the fractions of the short and long fluorescence lifetime, respectively, and their sum is 100 percent ( $\alpha_{1}+\alpha_{2}=1$ ). *τ*_*1*_, *τ*_*2*_ are the corresponding short and long lifetimes, and *C* accounts for background light. NAD(P)H has a short lifetime at the free conformation, and a long lifetime when bound to a protein. Conversely, FAD has a long lifetime when it is free, and a short lifetime when bound.

Images were then segmented into individual cell, cytoplasm, and nucleus compartments to acquire cell-based fluorescence lifetime endpoints. The cell segmentation process was based on the NAD(P)H intensity images and achieved in CellProfiler using a customized pipeline (Supplementary Material Appendix). Mitochondria masks were created by selecting the brightest 20% of pixels in each cell’s cytoplasm from the NAD(P)H intensity images to separate the lifetime values of mitochondria and cytosol. Image processing was performed using MATLAB to calculate the images of optical redox ratio (FAD fluorescence intensity divided by the summed intensity of FAD and NAD(P)H, FAD/(NAD(P)H + FAD)), weighted average fluorescence lifetime ( $\tau_{m}=\alpha_{1}\tau_{1}+\alpha_{2}\tau_{2}$ ), and fluorescence lifetime redox ratio (FLIRR, bound NAD(P)H fraction divided by the bound FAD fraction, NAD(P)H *α*_*2*_/FAD *α*_*1*_). Twelve NAD(P)H and FAD fluorescence features including optical redox ratio, FLIRR, NAD(P)H *τ*_*m*_, NAD(P)H *τ*_*1*_, NAD(P)H *τ*_*2*_, NAD(P)H *α*_*1*_, NAD(P)H intensity, FAD *τ*_*m*_, FAD *τ*_*1*_, FDA *τ*_*2*_, FAD *α*_*1*_, and FAD intensity were averaged across all pixels within a cytoplasm for each segmented cell for a single cell-level data.

### 2.4 Statistical analysis and classification

Data analysis was performed in R Studio. A two-sided Wilcoxon test with Bonferroni correction was used to indicate differences across cell groups for each fluorescence lifetime endpoint, and an alpha significance value of 0.05 was used to indicate significance. The Uniform Manifold Approximate and Projection (UMAP) method was used to visualize clustering within the autofluorescence imaging datasets (Becht et al., 2018). Cancer cells treated with sodium cyanide were defined as the OXPHOS inhibition group. Cells treated with 50 mM 2-DG and exposed to no glucose media were identified as the glycolysis inhibition group. Classical machine learning algorithms (random forest tree (RFT), support vector machine (SVM), quadratic discriminant analysis (QDA)) were trained to classify cells with inhibited glycolysis versus cells with inhibited OXPHOS based on the fluorescence lifetime features. The RFT model utilized 50 trees, comprising a total of 166 nodes. The minimum size of the terminal node was 1, and 5 variables were randomly sampled as candidates at each split. The supporting vector machine was configured as a classification machine, with training and prediction employing a linear kernel. These models were trained on a randomly selected 75% (1,365 cells) of the dataset and tested on the remaining 25% (454 cells) of the dataset. A receiver operation characteristic (ROC) curve and confusion matrix were used to evaluate the performance of the models on test datasets. The importance within the RFT classification model and the AUC (area under the curve) value of the ROC curve of each fluorescence lifetime feature were used to assess each feature’s contribution to the prediction. Each model was tested with 5-fold cross-validation and an average accuracy was computed to ensure its robustness. When predicting the metabolic activities of liver cancer cells, to avoid lifetime parameter differences within cell types, each feature value was normalized with the mean value of corresponding control cells to get the relative changes, and a new model was trained with normalized FLIM features.

### 2.5 Image preprocessing and CNN development

Since CNN development requires a larger dataset than the classical machine learning algorithms, the glycolysis and OXPHOS inhibition experiments were repeated to obtain ∼5,000 original cells (Supplementary Table S7). Each cell was extracted based on the bounding box of its mask generated by CellProfiler to produce six autofluorescence lifetime endpoint images (NAD(P)H *τ*_*1*_, NAD(P)H *τ*_*2*_, NAD(P)H *α*_*1*_, NAD(P)H *τ*_*m*_, NAD(P)H intensity, and FAD intensity). Then, the following image preprocessing procedure (Supplementary Table S17) was achieved in Python with the help of the OpenCV package to prepare for the training of CNN. A LeNet architecture for the CNN was developed using the machine-learning library Keras with a Tensorflow backend in Python running on Jupyter Notebook on the platform Anaconda 3. The input layer was adjusted to 40 × 40 with different channel numbers to fit the input number of lifetime components (Figure 5A). The loss function is the cross-entropy loss between true labels and predicted labels, which calculates the score that penalizes the probabilities based on the distance from the expected value, $\text{Loss}=-\frac{1}{\text{N}}\sum_{\text{i}=1}^{\text{N}}{\text{y}}_{\text{i}}\text{log}\hat{{\text{y}}_{\text{i}}}+\left(1-{\text{y}}_{\text{i}}\right)\text{log}(1-\hat{{\text{y}}_{\text{i}}}$ , where, $y_{i}$ is the actual values, and $\hat{y_{i}}$ is the neural network prediction. All CNN classifiers were trained to identify cells in glycolytic and oxidative phenotypes at a single-cell level. Further information about image preprocessing and CNN training can be found in the Supplementary Material Appendix.

### 2.6 Seahorse metabolic flux assay

A Seahorse XFe96 extracellular flux analyzer (Seahorse Biosciences, Santa Clara, CA) was used to assess the mitochondrial and glycolytic function of the cells in different metabolic groups. MCF7 breast cancer cells were plated at two densities (5 × 10^5^ cells/mL, 10^6^ cells/mL) on a Seahorse 96-well plate in a DMEM-based medium without phenol red, bicarbonate, glucose, pyruvate, or glutamine. Pyruvate (1 mM), glucose (10 mM), 2-DG (50 mM), and sodium cyanide (4 mM) were sequentially injected into the media. Oxygen consumption rate (OCR) and extracellular acidification rate (ECAR) were measured every 5 min for 15 cycles.

## 3 Results

### 3.1 NAD(P)H fluorescence lifetime imaging reveals glycolytic and OXPHOS states of cancer cells

**FIGURE 1:**
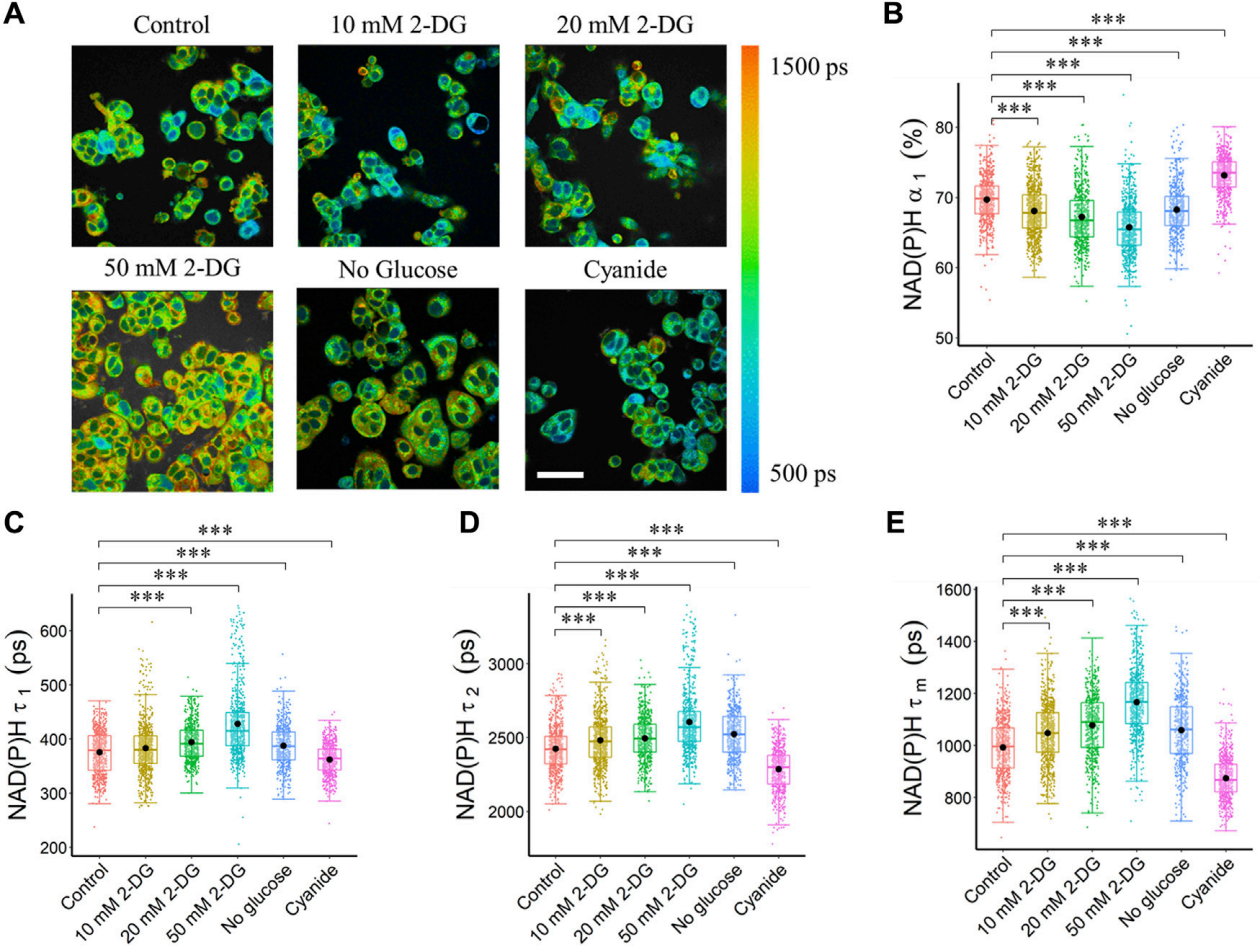
Glycolysis and OXPHOS inhibition alter the NAD(P)H fluorescence lifetimes of MCF7 cells. Glycolysis inhibition increased NAD(P)H fluorescence lifetime (*τ*_*1*_, *τ*_*2*_, *τ*_*m*_), and reduced free NAD(P)H fraction (*α*_*1*_), while OXPHOS inhibition reduced NAD(P)H fluorescence lifetime (*τ*_*1*_, *τ*_*2*_, *τ*_*m*_), and increased free NAD(P)H fraction (*α*_*1*_). **(A)** Representative NAD(P)H *τ*_*m*_ images, scale bar = 60 μm. **(B)** NAD(P)H *α*_*1*_ **(C)** NAD(P)H *τ*_*1*_ **(D)** NAD(P)H *τ*_*2*_ and **(E)** NAD(P)H *τ*_*m*_. ***p < 0.001 for two-sided Wilcoxon test with Bonferroni correction for multiple comparisons. Substrates in each media: Control (25 mM glucose +1 mM pyruvate), 2-DG (25 mM glucose +1 mM pyruvate +10/20/50 mM 2-DG), No glucose (50 mM pyruvate), Cyanide (25 mM glucose +1 mM pyruvate +4 mM NaCN).

In the autofluorescence images of NAD(P)H of MCF7 cells, the nuclei were darker than the cytoplasm because NAD(P)H was primarily located in the cytosol and mitochondria (Figure 1A). Metabolic perturbations to enhance and inhibit glycolysis altered autofluorescence lifetime features averaged across the cytoplasm pixels of each segmented cell (Supplementary Table S1, Figure 1). Inhibition of glycolysis within MCF7 cells with 2-DG treatment and glucose starvation resulted in a decreased NAD(P)H free fraction (*α*_*1*_), an increased free NAD(P)H fluorescence lifetime (*τ*_*1*_), and an increased bound NAD(P)H fluorescence lifetime (*τ*_*2*_) as compared with control MCF7 cells (Supplementary Table S1, Figures 1B–D). These NAD(P)H lifetime variations led to a longer NAD(P)H mean lifetime (*τ*_*m*_) of MCF7 cells with inhibited glycolysis (Figure 1E). Additionally, titrated concentrations of 2-DG promoted consistent reductions in NAD(P)H free fraction (*α*_*1*_) and increases in NAD(P)H fluorescence lifetimes (*τ*_*1*_ *, τ*_*2,*_ *τ*_*m*_). Conversely, an increased NAD(P)H free fraction (*α*_*1*_), and shorter free and bound NAD(P)H fluorescence lifetime (*τ*_*1*_ *, τ*_*2*_) were observed in cyanide-treated MCF7 cells, relative to the corresponding values of control cells (Supplementary Table S1, Figures 1B–D). These lifetime variations resulted in a shorter NAD(P)H mean lifetime (*τ*_*m*_) for MCF7 cells with OXPHOS inhibition (Figure 1E). With OXPHOS inhibition by cyanide, the NAD(P)H intensity of cancer cells increased, and the FAD intensity decreased, causing a decrease in the intensity redox ratio (IRR, FAD/(FAD + NAD(P)H)) after cyanide exposure (Supplementary Figure S1, Supplementary Table S1). Glucose starvation resulted in a significant decrease in both NAD(P)H and FAD intensities, generating a lower intensity redox ratio (Supplementary Figure S1, Supplementary Table S1). Glucose starvation altered the FAD fluorescence lifetime features of MCF7 cells resulting in an increased mean FAD lifetime (Supplementary Figure S2, Supplementary Table S1).

The effects of pyruvate and glucose, as well as 2-DG and cyanide treatments, on glycolysis and mitochondrial respiration of MCF7 cells were measured with a Seahorse analyzer. The ECAR increased when glucose was added, and decreased with 2-DG injection, while no changes were observed for the addition of pyruvate or cyanide (Supplementary Figure S3A). The OCR decreased with cyanide injection (Supplementary Figure S3B).

### 3.2 NAD(P)H fluorescence lifetime imaging reveals glutaminolysis perturbations within cancer cells

**FIGURE 2:**
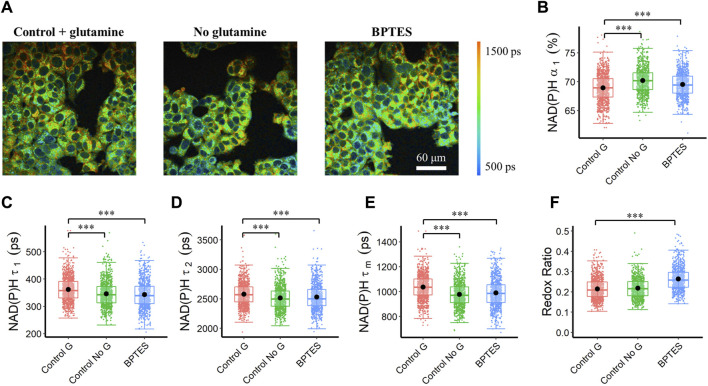
Autofluorescence lifetime variations of MCF7 cells in response to glutaminolysis inhibition. Glutaminolysis inhibition reduced NAD(P)H fluorescence lifetime (*τ*_*1*_, *τ*_*2*_, *τ*_*m*_), and increased free NAD(P)H fraction (*α*_*1*_). **(A)** Representative NAD(P)H *τ*_*m*_ images of control (Control G), no glutamine (Control No G), and BPTES-treated (BPTES) MCF7 cells, scale bar = 60 μm **(B)** NAD(P)H *α*_*1*_ **(C)** NAD(P)H *τ*_*1*_ **(D)** NAD(P)H *τ*_*2*_ **(E)** NAD(P)H *τ*_*m*_ **(F)** Intensity redox ratio (FAD/(FAD + NAD(P)H)). ***p < 0.001 for two-sided Wilcoxon test with Bonferroni correction for multiple comparisons. Substrates in each media: Control G (25 mM glucose +1 mM pyruvate +2 mM glutamine), Control No G (25 mM glucose +1 mM pyruvate), BPTES (25 mM glucose +1 mM pyruvate+ 2 mM glutamine +10 µm BPTES).

The stimulation and inhibition of the glutaminolysis pathway within MCF7 cells altered NAD(P)H fluorescence lifetimes. Inhibition of glutaminolysis with both glutamine starvation and BPTES treatment increased the NAD(P)H free fraction (*α*_*1*_), and decreased the free and bound NAD(P)H fluorescence lifetimes (*τ*_*1*_ *, τ*_*2*_) (Figures 2B–D), resulting in a decreased mean NAD(P)H lifetime (*τ*_*m*_) (Figures 2A,E). Furthermore, an increase in the intensity redox ratio was observed in the BPTES-treated cells compared with the control group (Figure 2F). Glutamine starvation increased the bound fraction of FAD (*α*_*1*_) and lowered the bound and free lifetimes (*τ*_*1*_, *τ*_*2*_) resulting in a lower mean FAD lifetime (*τ*_*m*_) (Supplementary Figure S4). BPTES treatment of MCF7 cells increased the lifetime of free FAD (*τ*_*2*_) but did not affect the other FAD lifetime parameters (Supplementary Figure S4C).

Autofluorescence lifetime imaging was performed on MCF7 cells fasted for 1 h and then exposed to 2 mM glutamine at 1, 2 and 3 h to isolate the effects of glutaminolysis from OXPHOS which proceeds once a cell has converted glutamine to α-ketoglutaric acid and to image at a quasi-steady state of metabolism following the addition of glutamine. An increase in NAD(P)H intensity and FAD intensity within the MCF7 cells was observed at 2 and 3 h of glutamate, as compared with the cells with glutamate for 1 h (Supplementary Figure S5). The NAD(P)H and FAD fluorescence lifetime components changed over time with glutamate stimulus. 1 h of glutamine stimulus increased the NAD(P)H lifetimes (*τ*_*1*_ *, τ*_*2,*_ *τ*_*m*_) and reduced the free fraction (*α*_*1*_) of NAD(P)H as compared with control MCF7 cells (Supplementary Figures S6A–D). Similarly, as compared with control MCF7 cells, 1, 2, and 3 h of glutamine stimulus increased the FAD lifetimes (*τ*_*1*_ *, τ*_*2,*_ *τ*_*m*_) and reduced the bound fraction (*α*_*1*_) of FAD (Supplementary Figures S6E–H).

### 3.3 Pyruvate concentration alters the FAD fluorescence lifetime

**FIGURE 3:**
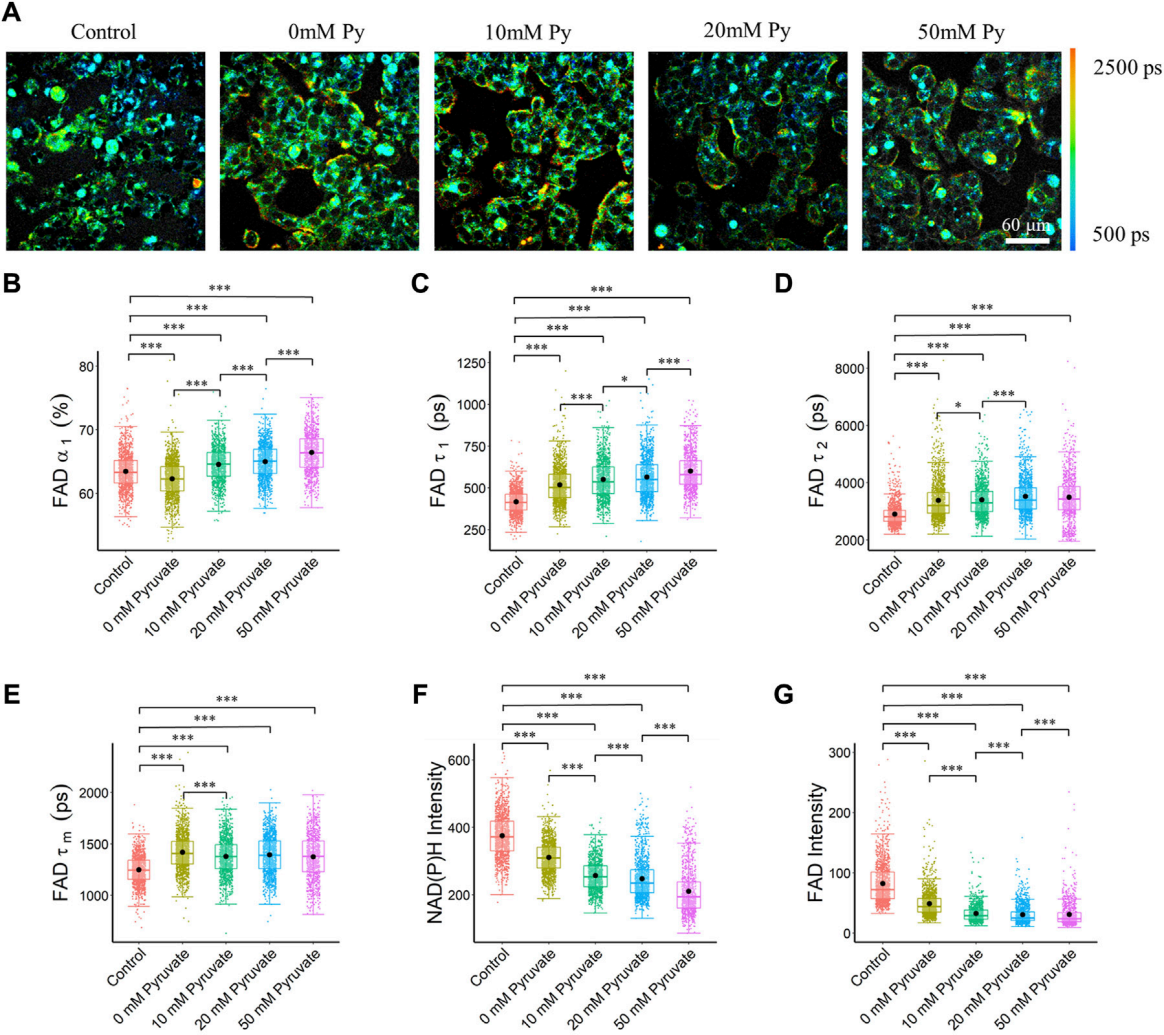
FAD fluorescence lifetimes of MCF7 cells were altered with different pyruvate concentrations of the culture media. Elevated pyruvate concentration resulted in an increase in the bound FAD fraction (*α*_*1*_), and bound FAD lifetime (*τ*_*1*_). **(A)** Representative FAD *τ*_*m*_ images of cancer cells exposed to different pyruvate concentrations, Py, pyruvate; scale bar = 60 μm **(B)** FAD *α*_*1*_ **(C)** FAD *τ*_*1*_ **(D)** FAD *τ*_*2*_ **(E)** FAD *τ*_*m*_ **(F)** NAD(P)H intensity **(G)** FAD intensity. *p < 0.05, ***p < 0.001 for two-sided Wilcoxon test with Bonferroni correction for multiple comparisons. Substrates in each media: Control (25 mM glucose +1 mM pyruvate), Pyruvate (no glucose + no glutamine +0/10/20/50 mM pyruvate).

To evaluate the effects of OXPHOS stimulation on autofluorescence lifetime metrics, MCF7 cells were fasted and then provided pyruvate at scaled concentrations. Cellular quantitation analysis showed that the pyruvate concentration groups had a longer bound (*τ*_*1*_) and free (*τ*_*2*_) FAD lifetime, which led to a longer mean FAD lifetime (*τ*_*m*_), as compared to the control group (Figures 3A, C–E). The cells exposed to different concentrations of pyruvate had an increased fraction of enzyme-bound FAD (*α*_*1*_) than the control cells (Figure 3B). Furthermore, pyruvate starvation caused a reduced fraction of bound FAD (*α*_*1*_), while increased pyruvate concentration increased the bound FAD fraction (*α*_*1*_), and bound FAD lifetime (*τ*_*1*_) (Figures 3B,C). A longer mean NAD(P)H fluorescence lifetime (*τ*_*m*_) of MCF7 cells can be observed in the pyruvate groups than in the control groups due to reduced free NAD(P)H fraction, and increased free and bound NAD(P)H lifetime (Supplementary Figure S7C–F). Additionally, the intensity of both NAD(P)H and FAD were lower in the pyruvate assay groups than in the control group, and an increased pyruvate concentration reduced both the NAD(P)H and FAD intensities, which led to reduced optical redox ratio (Figures 3F,G, Supplementary Figure S7B).

### 3.4 NAD(P)H and FAD fluorescence lifetime features predict cellular metabolic perturbations

**FIGURE 4:**
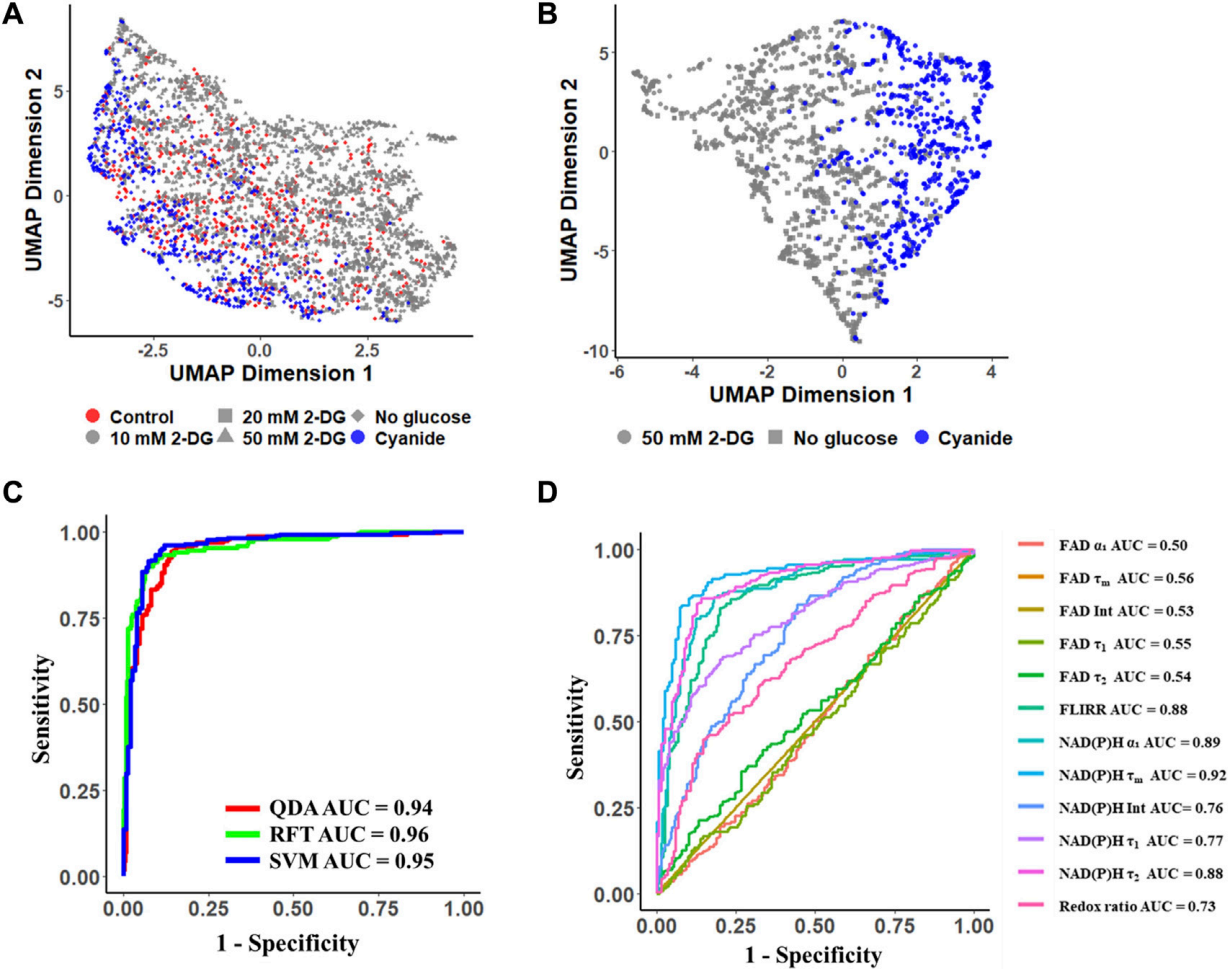
Autofluorescence lifetime features allowed classification of metabolic perturbations of MCF7 cells. Conventional machine learning models trained on autofluorescence lifetime features achieved approximately 90% accuracy in classifying glycolytic and oxidative cancer cells. **(A, B)** UMAP data reduction technique allows visual representation of the separation between OXPHOS maximum (glycolysis inhibition groups: 10 mM 2-DG, 20 mM 2-DG, 50 mM 2-DG, No glucose) and glycolysis maximum cells (OXPHOS inhibition groups: Cyanide). Each color represents a metabolic group, red corresponds to control, grey and blue to glycolysis inhibition, and OXPHOS inhibition respectively. Each shape represents a different drug-treated group. ROC curves of the test data for **(C)** machine learning classification models, RFT: random forest tree; SVM: support vector machine; QDA: quadratic discriminant analysis, and **(D)** lifetime features for classification of glycolytic versus oxidative cancer cells. Int, intensity; Redox ratio = FAD/(FAD + NAD(P)H); FLIRR = NAD(P)H *α*_*2*_/FAD *α*_*1*_.

UMAP visualization of the 12 autofluorescence imaging features (NAD(P)H *α*_*1*_ *, τ*_*1*_ *, τ*_*2*_ *, τ*_*m*_, intensity; FAD *α*_*1*_ *, τ*_*1*_ *, τ*_*2*_ *, τ*_*m*_, intensity; redox ratio; FLIRR) revealed the cluster separation of MCF7 cells with glycolysis inhibition groups (grey dots) from the OXPHOS inhibition groups (blue dots) with the control cell group (red dots) in the middle and overlapping with both inhibition groups (Figure 4A). A UMAP of the subset of data with MCF7 cells treated with 50 mM 2-DG and glucose starvation as the glycolysis inhibition groups, and treated with cyanide for maximizing the OXPHOS activities, showed a significant separation of MCF7 cells with inhibited glycolysis (grey dots) and cells with inhibited OXPHOS (blue points) (Figure 4B).

Using the MCF7 metabolic perturbation data, machine learning algorithms were developed to predict cellular metabolic variations, and the accuracy was calculated as the percentage of correctly classified cells over the total number of cells. A RFT model achieved a mean prediction accuracy of 92% and an ROC AUC value of 0.96 (Figure 4C, Supplementary Figure S8A). The support vector machine (SVM) model achieved a mean prediction accuracy of 90% and an ROC AUC of 0.95 (Figure 4C, Supplementary Figure S8B). The quadratic discriminant analysis (QDA) obtained a mean prediction accuracy of 92% and an AUC value of 0.94 (Figure 4C, Supplementary Figure S8C). When normalizing the data with the corresponding control cell group, the RFT model achieved a mean prediction accuracy of ∼0.97 with an ROC AUC value of ∼0.99 (Supplementary Figure S8D, E). Moreover, the separation of mitochondria and cytosol signals enhanced the prediction of metabolic phenotypes. The RFT model trained with cytosol lifetime values achieved an average accuracy of ∼90.5% in a 5-fold cross-validation and improved to approximately 94% using mitochondria lifetime values to discriminate between glycolytic and oxidative cells (Supplementary Figure S9).

Feature analysis from the RFT model revealed that the mean NAD(P)H lifetime (*τ*_*m*_) contributed most to this prediction, followed by the free NAD(P)H fraction (*α*_*1*_) (Supplementary Figure S10). Furthermore, the ROC AUC of models built from each feature individually implied that NAD(P)H *τ*_*m*_ (AUC = 0.92), and NAD(P)H *α*_*1*_ (AUC = 0.89), FLIRR (AUC = 0.88) and NAD(P)H *τ*_*2*_ (AUC = 0.88) were significant features for the prediction of cancer cell metabolic perturbations (Figure 4D). FAD lifetime features had low ROC AUC values and feature importance (Figure 4D, Supplementary Figure S10).

### 3.5 Convolutional neural networks can predict metabolic activities of MCF7 cells from NAD(P)H and FAD fluorescence lifetime images

**FIGURE 5:**
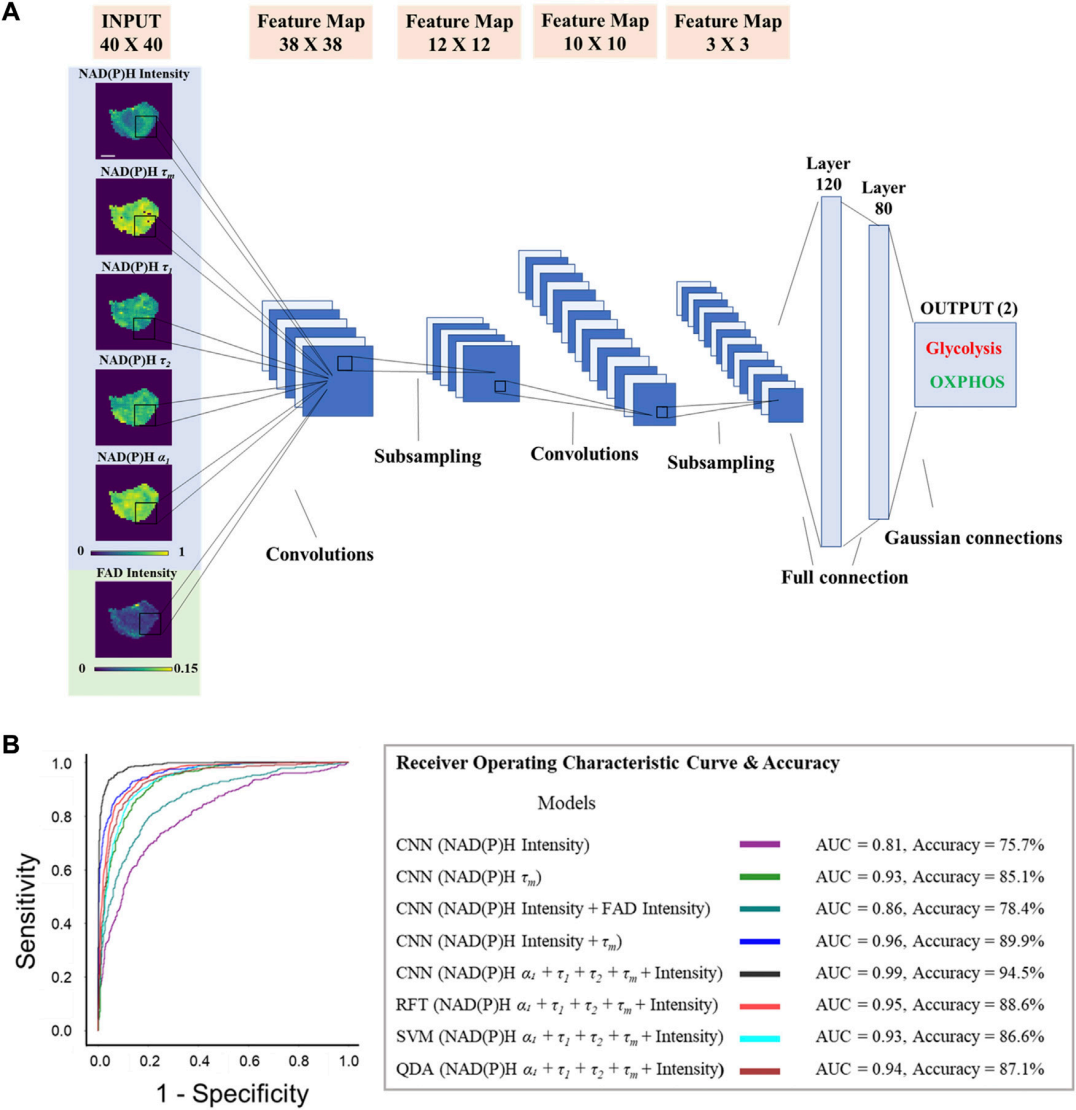
Metabolic activities of breast cancer cells are predicted from autofluorescence lifetime images. CNN models trained on autofluorescence lifetime images achieved approximately 95% accuracy in classifying glycolytic and oxidative cancer cells. **(A)** Predicting MCF7 metabolism as glycolysis inhibition or OXPHOS inhibition from autofluorescence lifetime images with CNN. The LeNet CNN models were trained with different fluorescence lifetime component images (NADH *τ*_*1*_, NADH *τ*_*2*_, NADH *α*_*1*_, NADH *τ*_*m*_, NADH intensity, FAD intensity), scale bar = 7 μm. **(B)** AUC ROC curves and accuracy for the test dataset (n = 1,520) of each classifier built to predict metabolism as glycolysis inhibition or OXPHOS inhibition. RFT: random forest tree; SVM: support vector machine; QDA: quadratic discriminant analysis.

A conventional LeNet CNN architecture was trained with different autofluorescence lifetime feature images and the training parameters were tuned to achieve the best performance (Figure 5A). Since no consistent difference was observed in the FAD lifetime between glycolytic and oxidative cells, and FAD lifetime values contributed less to the ML learning models, the CNN inputs were limited to FAD intensity images, as incorporating FAD lifetime images for training would require more memory and time for processing, without sufficient enhancement of model performance. The performance of each CNN model was assessed based on the prediction results of the test dataset. The prediction result was presented in a confusion matrix, where glycolysis inhibition cells were defined as the negative group, and the OXPHOS inhibition cells were defined as the positive group. The precision was determined as the percentage of true positives out of all the positive predictions. The recall was calculated as the percentage of correctly predicted positive cases out of the total actual positive cases.

The CNN models reached the least validation loss at ∼ 80 epochs with a 0.00001 learning rate (Supplementary Figure S11). When trained with only NAD(P)H intensity images, the LeNet CNN achieved an average test dataset accuracy of 75.7%, an ROC AUC of 0.81, a precision of 76.5%, and a recall of 60.0% for predicting glycolytic versus oxidative cells (Figure 5B, Supplementary Figure S12). Similar to the classical machine learning model results, including FAD intensity images did not significantly improve this prediction. When the CNN model was trained on both NAD(P)H intensity and FAD intensity images together, the accuracy of the test data increased by ∼3% (78.4%) with an ROC AUC value of 0.86 (Figure 5B, Supplementary Figure S12). The CNN model performed better when trained with NAD(P)H lifetime images, as compared to the intensity images. The CNN model trained with NAD(P)H *τ*_*m*_ images classified glycolytic MCF7 cells from oxidative cells with an average accuracy of 85.1%, a precision of 87.4%, a recall of 81.3%, and an ROC AUC of 0.93 for the test data (Figure 5B, Supplementary Figure S12). Including additional NAD(P)H lifetime component images in the CNN improved the accuracy, and the highest performance achieved for the CNN model was trained with all NAD(P)H lifetime components (intensity, *τ*_*1*_, *τ*_*2*_, *τ*_*m*_, *α*_*1*_). This model achieved 94.5% accuracy, 0.99 ROC AUC, 98.2% precision, and 91.5% recall. (Figure 5B, Supplementary Figure S12). The performance of the LeNet CNN trained with images of all NAD(P)H lifetime components (intensity, *τ*_*1*_, *τ*_*2*_, *τ*_*m*_, *α*_*1*_) exceeded the prediction performance of classical machine learning algorithms, including RFT, SVM, and QDA, trained and tested with the same datasets (Figure 5B, Supplementary Figure S12).

### 3.6 The metabolic prediction models transfer to additional datasets

To further access the applicability of the fluorescence lifetime model to predict metabolic pathways, the models were first tested with data from the 10 mM and 20 mM 2-DG concentration groups, and the pyruvate titration data. Using the classical machine learning model, more than 80% of the MCF7 cells exposed to 10 mM 2-DG and 20 mM 2-DG were predicted to have glycolysis inhibition (Supplementary Table S2). When tested with the pyruvate assay data, where MCF7 cells were only provided pyruvate as a substrate, more than 95% of the cells were predicted to have glycolysis inhibition by the classical machine learning model (Supplementary Table S2). Furthermore, the classical machine learning model predicted that over 95% of the cells fed with glutamine rely more on the oxidative pathway (Supplementary Table S2).

**FIGURE 6:**
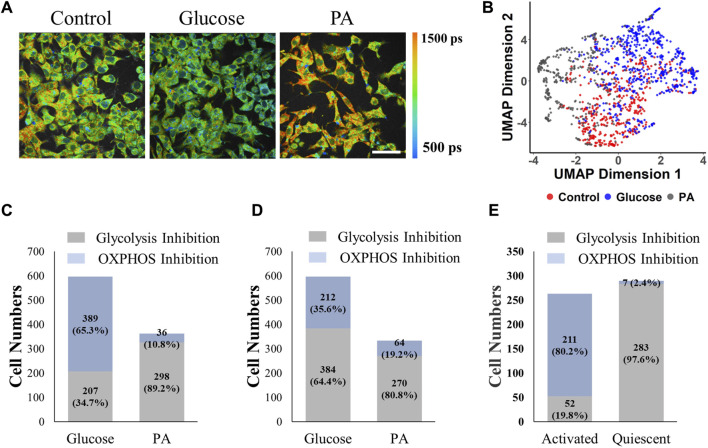
MCF7 cell-trained model prediction performance for liver cancer cells and T cells. The RFT machine learning model trained with normalized MCF7 cell data effectively predicted glycolysis or OXPHOS use in hepatocellular cells, and T cells. **(A)** Representative NAD(P)H *τ*_*m*_ images of HepG2 cells exposed to control media (starved condition), glucose (30 mM), and palmitate (0.4 mM PA), scale bar = 60 μm **(B)** UMAP data reduction technique allows visual representation of the separation between different metabolic groups of hepatocellular cells. Each color represents a metabolic group, red corresponds to control, grey and blue to glucose treated, and PA treated respectively. **(C)** Prediction of HepG2 cell metabolism by the RFT model trained with MCF7 cells. **(D)** Prediction of HepG2 cell metabolism by the CNN model trained with MCF7 cells. **(E)** Prediction of activated and quiescent T cell metabolism by the RFT model trained with MCF7 cells.

Finally, the models trained with MCF7 cells were evaluated to identify metabolic variations of other cells. Glucose-treated HepG2 liver cancer cells had more fraction of free NAD(P)H (*α*_*1*_), and shorter NAD(P)H lifetimes (*τ*_*m*_) than the control cells (Figure 6A, Supplementary Figure S13A, D). Palmitate treatment of HepG2 cells caused a lower fraction of free NAD(P)H (*α*_*1*_), and longer free and bound NAD(P)H lifetimes (*τ*_*1*_, *τ*_*2*_) compared to the control cells (Supplementary Figure S13A–C). When adapting the UMAP algorithm to visualize distributions of different cellular groups, a separation between cells exposed to glucose and cells exposed to palmitate (PA) was observed in Figure 6B. When applying the RFT machine learning model trained with normalized MCF7 cell data, 65.4% of the glucose-treated cells were predicted to be glycolytic (OXPHOS inhibition), and 89.2% of the palmitate exposed cells were predicted to be oxidative (glycolysis inhibition) (Figure 6C). When applying the CNN model (trained with all NAD(P)H lifetime features) that was trained with the MCF7 cells to the HepG2 liver cancer data, 80.8% of the palmitate exposed cells were predicted to have glycolysis inhibition which was consistent with the prediction of the classical machine learning model (Figure 6D). In contrast, 64.4% of the glucose-exposed cells were predicted to be glycolysis inhibition (Figure 6D). Moreover, MCF7 metabolic prediction models were also tested with FLIM data from primary human T cells, which have different metabolic phenotypes by activation state. Activated T cells are more dependent on glycolysis and quiescent T cells are more dependent on OXPHOS. The ratio of OCR to ECAR was significantly decreased in activated T cells compared with that of quiescent T cells (Walsh et al., 2021). Using the RFT model, 80.2% of the activated T cells were predicted to be glycolytic (OXPHOS inhibition), and 97.6% of the quiescent T cells were predicted to be oxidative (glycolysis inhibition) (Figure 6E). However, most (98+%) of both the activated and quiescent T cells were predicted to exhibit OXPHOS inhibition with the CNN model (Supplementary Table S3).

## 4 Discussion

Autofluorescence lifetime imaging is sensitive to metabolic differences in live cells between groups such as cancer and non-cancer cells, phenotypes of immune cells, and stem cells and differentiated cells (Bird et al., 2005; Skala et al., 2007; Ostrander et al., 2010; Quinn et al., 2013; Walsh et al., 2013; Varone et al., 2014; Palmer et al., 2015; Alfonso-Garcia et al., 2016; Heaster et al., 2020; Walsh et al., 2021). By using endogenous fluorophores for contrast, autofluorescence lifetime imaging resolves cellular and sub-cellular resolution without contact or manipulation of the sample providing label-free advantages and independence from label-related confounding factors. However, autofluorescence measurements lack the specificity provided by protein- or molecule-targeted labels. Selection of specific excitation and emission wavelengths can isolate endogenous fluorophores such as NAD(P)H and FAD (Huang et al., 2002). Two-component exponential decay fitting of NAD(P)H and FAD fluorescence lifetimes allows quantification of the fraction of bound or free coenzymes, and the lifetime values of the short and long lifetimes (Nakashima et al., 1980; Lakowicz et al., 1992). Despite this biochemical specificity, an interpretation or relationship between NAD(P)H and FAD fluorescence lifetime metrics and macroscopic cellular metabolic phenotypes remains elusive. In this study, metabolic perturbation experiments were designed to selectively activate or inhibit the metabolic pathways of glycolysis, OXPHOS, and glutaminolysis via controlled substrate availability and metabolic inhibitors. Then, NAD(P)H and FAD fluorescence lifetime images were acquired and analyzed to define fluorescence lifetime features of cells using specific metabolic pathways. Finally, this robust dataset of autofluorescence lifetime data was used to design and evaluate models for predicting cellular use of glycolysis or OXPHOS. The results show that fluorescence lifetime imaging of NAD(P)H and FAD allows a non-destructive technique to predict metabolic perturbations of cancer cells when treated with inhibitors or exposed to culture environment variations at the single-cell level.

Cancer cells often have enhanced glycolysis, even in the presence of oxygen and uncompromised mitochondrial function, to promote growth, proliferation, and survival (Warburg, 1956). 2-DG (2-deoxy-D-glucose) is a glucose analog that competitively inhibits glycolysis by binding to the enzyme hexokinase, and cyanide binds to cytochrome c oxidase and prevents the transfer of electrons to oxygens in OXPHOS. Changes in metabolic pathways within MCF7 cells alter the fluorescence lifetimes of NAD(P)H and FAD. The reduction of free NAD(P)H fraction (*α*_*1*_) with 2-DG treatment was observed in MCF10A breast cancer cells and pancreatic islet cells (Drozdowicz-Tomsia et al., 2014; Wang et al., 2021). Conversely, a higher level of glycolysis induced more fraction of free NAD(P)H in kidney cells, neural cells, and stem cells in disease states (Stringari et al., 2012a; Chakraborty et al., 2016; Sameni et al., 2016). Herein, glycolysis inhibition and OXPHOS inhibition in the cancer cells resulted in the exact opposite changes in NAD(P)H lifetime features (Figure 1, Supplementary Table S1), and the consistent and opposite changes in NAD(P)H fluorescence lifetimes support the hypothesis that NAD(P)H fluorescence lifetimes are responsive to metabolic pathway shifts and changes in the protein binding partners of NAD(P)H (Liu et al., 2018; Sharick et al., 2018). Consequently, NAD(P)H lifetime features (*τ*_*m*_, *α*_*1*_ *, τ*_*2*_) contributed significantly to the prediction of glycolytic versus oxidative cells (Figure 4D, Supplementary Figure S10). Especially, NAD(P)H *α*_*1*_ is the highest weighted feature for the classification of T cell activation, allowing robust differentiation of oxidative quiescent T cells from glycolytic, activated T cells (Walsh et al., 2021).

In contrast to the opposite variations observed in NAD(P)H lifetime features of MCF7 cells with isolated glycolysis and OXPHOS metabolism, no consistent changes in the FAD lifetime were observed with direct glycolysis and OXPHOS perturbations with cyanide, glucose-starvation, and 2-DG treatment (Supplementary Figure S2, Supplementary Table S1). However, published studies revealed FAD lifetime changes in lung cancer cells exposed to rotenone/antimycin for OXPHOS inhibition, which resulted in a reduced free-to-bound fraction of FAD (Penjweini et al., 2020). Additionally, contradictory variations of FAD fluorescence lifetime have been observed in bladder cancer cells, skin cells, stem cells, and neural cells due to shifts between glycolysis and OXPHOS (Chakraborty et al., 2016; Meleshina et al., 2017; Becker et al., 2020; Ung et al., 2021). The contradictory lifetime results of FAD autofluorescence may be due to contributions of additional flavins to autofluorescence images. Flavin molecules including flavin mononucleotide (FMN) and riboflavin can contribute to the observed FAD fluorescence, introducing potential confounding factors when using FAD fluorescence to study metabolism. While the emission of FAD and free FMN both peak around 530 nm, protein-bound FMN peaks around 495 nm. Although FMN contributions to flavin autofluorescence imaged with a 542–582 nm emission filter were estimated to be ∼5% (Kalinina et al., 2021), differences in emission filters may alter the composition of the flavin autofluorescence. For example, a blue-shifted filter may increase the contribution of protein-bound FMN and reduce the sensitivity of the flavin autofluorescence measurements to FAD, confounding the interpretation of flavin autofluorescence lifetime data. Although FMN is typically only a small portion of the flavin autofluorescence for emission filters centered around 550 nm, protein-bound FMN has a longer fluorescence lifetime (around 5 ns) than FAD (van den Berg et al., 2002), and changes in FMN concentration or binding dynamics could alter the measured lifetime values (Reichert et al., 2023).

**FIGURE 7:**
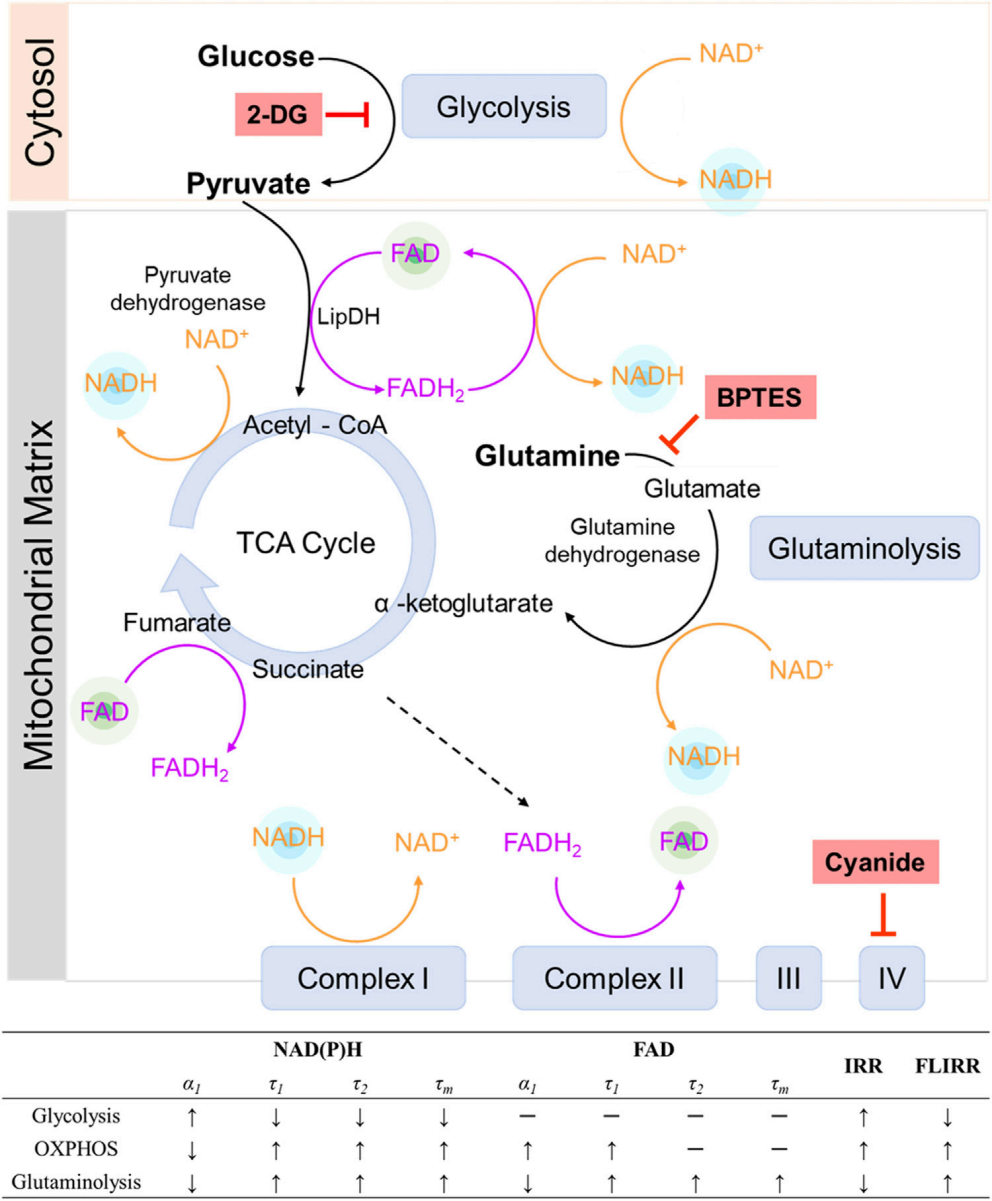
The roles of NADH and FAD in metabolic pathways. NADH and FAD are coenzymes in glycolysis, the TCA cycle, glutaminolysis, and the electron transport chain. 2-DG inhibits glycolysis, forcing a cell to use alternative pathways for metabolism. Cyanide inhibits complex IV of the electron transport chain, effectively inhibiting OXPHOS. BPTES inhibits glutaminolysis. Each metabolic pathway varies the fluorescence lifetimes of NAD(P)H and FAD differently. IRR = intensity redox ratio FAD/(FAD + NAD(P)H), FLIRR = fluorescence lifetime redox ratio NAD(P)H *α*_*2*_/FAD *α*_*1*_.

The use of FAD as a primary electron carrier at multiple steps throughout the TCA and the electron transport chain suggests that FAD fluorescence lifetimes should be sensitive to alterations in cellular use of the oxidative metabolic pathway. To further evaluate the role of OXPHOS on FAD fluorescence lifetimes, MCF7 cells were grown and imaged in media with titrated concentrations of pyruvate and an absence of other metabolic substrates. Pyruvate enters the mitochondria and is converted to acetyl-CoA by pyruvate dehydrogenase (PDH) to facilitate oxidative phosphorylation (Figure 7). In this process, pyruvate dehydrogenase (PDH) acts as a gatekeeper between glycolysis and oxidative phosphorylation, and FAD is reduced to FADH_2_ through lipoamide dehydrogenase (LipDH) (Figure 7). FADH_2_ can then be oxidized to reduce NAD^+^ to NADH, which contributes to the electron transport chain. The optical redox ratio ((FAD/(NAD(P)H + FAD)) was reduced at 10, 20, and 50 mM pyruvate (Supplementary Figure S7B), which implies an increased oxidative state within the cells (Varone et al., 2014). The cancer cells exhibit a higher proportion of bound FAD (*α*_*1*_) as the concentration of pyruvate in the media is increased, as compared to the control cells (Figure 3B), suggesting an expected increased use of FAD due to increased oxidative metabolism and throughput in the TCA cycle, where FAD catalyzes the oxidation of succinate into fumarate (Figure 7) (Martinez-Reyes and Chandel, 2020).

Elevated levels of glutaminolysis have also been found in cancer cells to compensate for changes in glycolysis and maintain a functional TCA cycle (Lj Reitzer and Kennell, 1979; Medina, 2001; Wise and Thompson, 2010). Glutamine is converted to glutamate by enzyme glutaminase, and further metabolized to α-ketoglutarate by glutamate dehydrogenase and fuels the TCA cycle, while BPTES specifically targets glutaminase to inhibit glutaminolysis (Figure 7). In glutaminolysis, NAD^+^ is reduced to NADH and contributes to the mitochondrial-bound NADH pools (Varone et al., 2014) (Figure 7). An increased NAD(P)H fluorescence intensity in the fasted cells was observed at 1, 2, and 3 h after glutamine stimulation (Supplementary Figure S5A), consistent with the expectation that glutaminolysis increases NADH. Furthermore, the contribution of glutaminolysis to the bound NAD(P)H fraction in MCF7 cells (Figure 2B) is consistent with prior published results in human foreskin keratinocytes and C2C12 myoblasts (Liu et al., 2018). Glutaminolysis is known to be coupled with oxidative phosphorylation (OXPHOS) by fueling the tricarboxylic acid (TCA) cycle. This relationship is supported by the result that more than 95% of cells supplemented with glutamine were predicted to be oxidative phenotypes with the classical machine learning model (Supplementary Table S2), indicating that they rely on OXPHOS to generate ATP. Furthermore, glycolysis and glutaminolysis elicit opposite changes in structural metabolic readouts, including mitochondrial clustering and network analysis (Ghose et al., 2013; Xylas et al., 2015; Liu et al., 2018), and opposite trends of NAD(P)H fluorescence lifetime metrics of MCF7 were observed in cancer cells between glycolysis and glutaminolysis stimulation (Figures 1, 2, 7). The inhibition of glutaminolysis by BPTES has been found to decrease ATP levels and profoundly increase ROS levels (Le et al., 2012), which leads to changes in the optical redox ratio in cancer cells (Figure 2F).

Even though the lifetime values can change based on experimental factors like filters and fitting methods, unlike fluorescence intensity imaging, fluorescence lifetime imaging is a self-referenced measurement, and theoretically independent of laser power, detector sensitivity, and fluorophore concentration when only one fluorophore species is contributing to the decay. Therefore, FLIM provides a more robust technique than fluorescence intensity imaging. Indeed, the lifetime measurement of certain molecules in solution has been successfully reproduced across multiple configurations, providing a reliable calibration reference for fluorescence lifetime imaging (QS et al., 2001; Kristoffersen et al., 2014). Additionally, fluorescence lifetime imaging also offers information regarding binding status and microenvironment of the fluorophores, which can be used to infer additional biochemical information about NAD(P)H and FAD. Therefore, the FLIRR was created based on protein-bound variations of NAD(P)H and FAD during OXPHOS, to overcome the measurement-dependent limitations of the traditional intensity-based redox ratio (IRR), and provide metabolic information within a single metric (Cao et al., 2019; Penjweini et al., 2020). Furthermore, other formats of FLIRR have been extended using different lifetime components of NAD(P)H and FAD to define differences between tumor and normal cells (Kalinina et al., 2021). The FLIRR is sensitive to drug-induced metabolic alterations of human keratinocytes, prostate, and squamous cancer cells (Alam et al., 2017; Wallrabe et al., 2018; Cao et al., 2019; Kalinina et al., 2021). FLIRR reflects the protein-bound ratio of NAD(P)H and FAD and is not correlated with IRR across a variety of metabolic states in T cells (Hu et al., 2020). Additionally, the correlation of bound NAD(P)H fraction and FLIRR values has the potential to resolve subcellular information (Cao et al., 2019; Hu et al., 2020).

Due to the unique autofluorescence lifetime phenotypes observed for glycolytic or oxidative MCF7 cells, different models for predicting cellular metabolic phenotypes from the NAD(P)H and FAD fluorescence lifetime imaging data were compared. Machine learning models are appropriate for analysis of datasets with multiple variables, and have been used to extract and interpret cell phenotypes from fluorescence lifetime data. Several studies have applied extracted lifetime features with machine learning algorithms to identify mouse embryo health, quantify precancer cells, classify T cell activation, differentiate stem cell phenotypes, and investigate metabolic perturbations (Gu et al., 2015; Liu et al., 2018; Ma et al., 2019; Wang et al., 2020; Hu et al., 2021; Qian et al., 2021; Walsh et al., 2021). Here, both conventional machine learning methods and neural networks were trained with autofluorescence lifetime images and features to predict metabolic states of cancer cells. The performance of the models is comparable with published papers that use NAD(P)H intensity images or autofluorescence lifetime features to predict cell phenotypes (Liu et al., 2018; Wang et al., 2020; Qian et al., 2021). Normalization with the mean lifetime values of the control group improves classification accuracy (0.97), suggesting heterogeneity within the cancer cells or across technical replicates can be reduced by normalization (Supplementary Figure S8D, E).

Analyzing the spatial information of NAD(P)H and FAD fluorescent signals can improve metabolic studies. Firstly, the mitochondrial network structure is associated with metabolism and cellular function (Hackenbrock, 1968; C R Hackenbrock et al., 1971; Capaldi, 2004; Benard et al., 2007; Jheng et al., 2012). Since NAD(P)H is primarily localized in mitochondria and thus mitochondria appear as bright pixels in NAD(P)H images, analyzing mitochondrial organization from autofluorescent images offers complimentary morphology information, and reflects alterations in metabolic activities (Levitt et al., 2007; Dimitra Pouli et al., 2016). Specifically, the mitochondria are observed to be more clustered with a higher glycolytic level and less clustered when glutaminolysis and OXPHOS are dominant in tissues and cells (Xylas et al., 2015; Liu et al., 2018). Furthermore, spatial mapping of NAD(P)H and FAD lifetime metabolic activity can help resolve metabolic pathways, as glycolysis and OXPHOS occur in different cellular compartments. Using isolated lifetime values for potential mitochondria (pixels with higher NAD(P)H intensity) improved the accuracy of predicting glycolysis and OXPHOS in machine-learning models by 4% (Supplementary Figure S9). However, accurate mitochondria segmentation of cancer cells in fluorescence lifetime images remains challenging, as it requires high spatial resolution that may be compromised when using spatial binning during lifetime analysis. Convolutional neural networks (CNNs) are effective in retaining spatial information within the cell, enabling the identification of mitochondria and cytosol and facilitating metabolic activity studies. CNNs capture spatial relationships between different pixels in the cells through convolutional operations on input images and then downsample the feature maps using pooling layers to select the most significant values in the cellular regions. Previously, the LeNet CNN model achieved around 89% accuracy in classifying T cell activation when trained with around 8000 NAD(P)H intensity images (Wang et al., 2020). The best CNN model was achieved by training with all NAD(P)H 2D fluorescence lifetime component images together and achieved 95% accuracy and an AUC of 0.99 for the testing dataset, which exceeds the performance of conventional machine learning models that use extracted FLIM features (Figures 4, 5; Supplementary Figure S12).

To ensure the utility of the metabolism-prediction models for additional cells and studies beyond MCF7 cells, the models trained with breast cancer cells were tested on different samples. For liver cancer cells, palmitate treatment triggers fatty acid oxidation, which produces acetyl-CoA and contributes to the TCA cycle and electron transport chain. Palmitate has been observed to reduce the NAD(P)H bound fraction in myoblast cells, which is in contrast to cancer cells (Liu et al., 2018). Even though there are multiple metabolic pathways besides OXPHOS in the cancer cells with PA treatment, the pre-trained models only predict whether glycolysis or oxidation is the major metabolic activity. The pre-trained CNN model did not provide high accuracy for prediction of the metabolic phenotypes of liver cancer cells and T cells (Figure 6D, Supplementary Table S3), implying that the difference in morphological features such as cell size, and subcellular structure influence the performance of the CNN significantly. In contrast, the conventional machine learning models that use averaged FLIM features for each cell rather than the cell images, worked well to predict glycolysis or OXPHOS use by hepatocellular cells, suggesting that the autofluorescence endpoints reflect changes in metabolic variations that are consistent across these two different cancer cell types (Figure 6C). Furthermore, the RFT model, trained on MCF7 data, accurately predicted the metabolic shift of T cells from oxidative phosphorylation to glycolysis and glutaminolysis upon activation (Figure 6E) (Wang and Green, 2012; Chang et al., 2013). This finding is consistent with previous Seahorse results, which showed a significant decrease in the OCR to ECAR ratio in activated T cells compared to quiescent T cells (Walsh et al., 2021). Specifically, the T cell data was collected on a different microscope at a different location, indicating that the metabolic prediction model can be used across cell types, phenotypes, and instrumentation. The applicability of the MCF7-cell trained RFT model to accurately predict metabolic phenotypes across other cells and metabolic perturbations supports the promotion of ML-FLIM for identifying cellular metabolism in extensive research fields.

It is important to acknowledge that cells can exhibit multiple metabolic activities simultaneously, which are not mutually exclusive, and can switch between different metabolic pathways based on their energy requirements and nutrient availability. While it would be interesting to characterize glycolysis and OXPHOS on a continuous spectrum, this is limited by the lack of ground truth data for training and testing such models at a cellular level. Although machine learning models can output the predictive probability of cell assignment to each metabolic group (Supplementary Figure S14) and such information may encode relative contributions of the two metabolic pathways, the biological significance of this information requires further validation. Here, the impact of environmental factors that can confound metabolic analyses were minimized by well controlled studies of cultured cells. Future research will evaluate the models in complex environments to improve the applicability of these findings in both *in vivo* and *ex vivo* settings and investigate the potential of CNN models to incorporate additional metabolic pathways, further enhancing the ability to use autofluorescence lifetime imaging to evaluate cellular metabolic activities. Furthermore, mitochondria typically exhibit higher NAD(P)H intensity than the cytosol, making segmentation a promising approach to help discriminate glycolysis and OXPHOS based on their distinct spatial locations. While the difference in lifetime values between different metabolic groups was similar when analyzing mitochondria and cytosol regions (Supplementary Figures S15, 16), using isolated lifetime values from mitochondria showed a better performance of predicting glycolysis and OXPHOS in the machine learning models (Supplementary Figure S9B). Therefore, the importance of spatial information in discriminating metabolic activities is highlighted by the results of both ML and CNN models. Future technological developments that permit higher spatial resolution imaging and analysis of fluorescence lifetime imaging may further enhance spatially-dependent metabolic analysis.

## 5 Conclusion

In this paper, unique NAD(P)H and FAD lifetime alterations were observed within cancer cells with different levels of glycolysis, OXPHOS, and glutaminolysis. These alterations are sufficient for machine learning models to predict the predominance of glycolysis versus OXPHOS from the lifetime features and images. The FLIM image-based CNN models provided increased accuracy over traditional feature-based machine learning models due to spatial information within the cell, but are less transferrable to other cells. The RFT model trained with MCF7 cells can accurately predict the dominant metabolic pathway of liver cancer cells exposed to different metabolic environments and the metabolic status of activated or quiescent T cells. In summary, autofluorescence lifetime imaging offers a label-free, quantitative method to identify metabolic activities in living cells, and can be used across various platforms for broad applications to study metabolic changes due to chemotherapy, gene expression, and immune responses.

## Data Availability

The datasets presented in this study can be found in online repositories (https://github.com/walshlab/CancerCellMetabolism/tree/main). All data and code in this study are available from the corresponding author upon reasonable request.
