# Supplementary material for: Label-free spatially maintained measurements of metabolic phenotypes in cells
Source: Front Bioeng Biotechnol. 2023 Nov 28;11:1293268. doi: 10.3389/fbioe.2023.1293268 (PMC10715269; doi:10.3389/fbioe.2023.1293268)
Supplement: Supplementary file 1 [file DataSheet1.docx]

Label-free spatially maintained measurements of metabolic phenotypes in cells

Linghao Hu^1^, Nianchao Wang^1^, Joshua D Bryant^2^, Lin Liu^3, 4^, Linglin Xie^3^, A Phillip West^2, 5^, Alex J. Walsh^1*^

^1^Department of Biomedical Engineering, Texas A&M University, College Station, Texas, USA

^2^Microbial Pathogenesis& Immunology, Health Science Center, Texas A&M University, College Station, Texas, USA

^3^Department of Nutrition, Texas A&M University, College Station, Texas, USA

^4^Department of Integrative Physiology, Baylor College of Medicine, One Baylor Plaza, Houston, TX, USA

^5^The Jackson Laboratory, 600 Main Street, Bar Harbor, ME 04609, USA

*** Correspondence:**Alex J. Walsh
walshaj@tamu.edu

Supplementary Material

# Supplementary Information

## Metabolic Perturbation Experiment of Breast Cancer Cells

For glucose and OXPHOS inhibition experiments, two types of culture media were prepared for different treatments: control media 1 (CM1): DMEM (Gibco™, 10313021) with high glucose (25 mM) and pyruvate (1 mM), but without glutamine; and control media 2 (CM2) glucose starvation media: DMEM (Gibco™, A1443001) without glucose, glutamine, or pyruvate. Different concentrations of 2-Dexoy-D-glucose (10 mM, 20 mM, 50 mM, Thermo Scientific, AC111980050) were added to cells in the CM1 1 hour before imaging to allow reactions to inhibit the glycolysis pathways (Drozdowicz-Tomsia et al., 2014). For a glucose depletion group, MCF7 cells were seeded on imaging dishes in CM1 as described and the media was exchanged for CM2 supplemented with pyruvate (50 mM, Gibco™, 11360070) 1 hour before imaging. For the sodium cyanide (NaCN) group, cells were seeded in CM1 and NaCN (4 mM, SIGMA-ALDRICH, 380970) was added to CM1 to inhibit OXPHOS 5 minutes before imaging (Walsh et al., 2013). In the pyruvate concentration imaging experiment, CM2 was supplemented with titrated concentrations of pyruvate (0 mM, 10 mM, 20 mM, 50 mM). Cells were exposed to the media with different concentrations of pyruvate for 1 hour before imaging. For targeting glutaminolysis, three types of culture media were prepared for different treatments: control media 3 (CM3): DMEM with glucose (25 mM) and pyruvate (1 mM), and glutamine (2 mM, Gibco™, 25030081), glutamine depletion media: DMEM with glucose (25 mM) and pyruvate (1 mM), but without glutamine, and glutamine-only media: DMEM with glutamine (2 mM), but without glucose or pyruvate. To inhibit glutaminolysis, bis-2-(5-phenylacetamido-1,3,4-thiadiazol-2-yl) ethyl sulfide (BPTES, 10 µm, ASTATECH, A11656) was added to the cells in CM3 1 hour before imaging (Walsh et al., 2021). To isolate the glutaminolysis pathway over time, cells were plated in CM3 and the media was exchanged for the glutamine-only media, and the cells were imaged at 1, 2, and 3 hours. The concentration of metabolic substrates in each group is summarized in Table S4.

## Cell Segmentation Workflow

The cell segmentation was realized from the NAD(P)H intensity image using CellProlifer with a customized pipeline. Firstly, the raw NAD(P)H intensity images were rescaled between 0 and 1. Next, the backgrounds were extracted by using default object identification in CellProfiler. Since the nuclei in the cells were darker compared with surrounding cytoplasmic regions, the image features of speckles and dark holes were enhanced to improve the appearance of nucleus regions. Then an adaptive Otsu method was applied to identify the nucleus within a typical diameter range (5~20 for MCF7 cells), and the diameter can be adjustable for different cells. To distinguish between individual nuclei that are touching each other, each object was identified by the peak of brightness, and the dividing line was determined by the occurrence of indentation. Then, the cellular regions were identified by propagating from the pre-identified nucleus regions through an adaptive Otsu method. The threshold correction factor and size of the adaptive window were adjusted to optimize the performance of the Otsu method in different cells. Cytoplasm masks were determined by subtracting the nucleus objects from cell objects. Finally, all identified cells were filtered based on the area of the cytoplasm to exclude clumped cells and error objects. The cell number of each group is summarized in Supplementary Table S5 and Table S6.

## Image Preprocess Routine

The image preprocess was achieved in Python running on Jupyter Notebook on the platform of Anaconda3 with the help of the OpenCV package. To remove incomplete or uninformative cells, the cells were filtered by thresholding the entropy of NAD(P)H intensity and NAD(P)H *τ_m_* images, and the threshold values were defined according to the distribution of entropy with a Gaussian approximation. Since the CNN classifier requires inputs of uniform size, we padded all cancer cells to be 40 x 40 pixels with black borders. The padding size was decided by the largest cell size and the cell size distribution of the dataset. After noise removal and padding, a montage of images of the cells in each metabolic group was visually inspected. Finally, the original training dataset was augmented by rotating each cell image by 45, 90, 135, 180, and 270 degrees, and also by flipping the cell image horizontally and vertically. This procedure amplified the size of the original training dataset by seven times (Table S7). To test the metabolic prediction performance of different autofluorescence lifetime features by CNN, input datasets consisted of all NAD(P)H fluorescence lifetime components (NADH *τ_1_*, NADH *τ_2_*, NADH *α_1_*, NADH *τ_m_*, NADH intensity, FAD intensity) or a subset of these images (Figure 5).

## CNN Training and Evaluation

The LeNet model was composed of two convolutional layers and two pooling layers (Y LeCun, 1990). The input layer was adjusted to 40 x 40 with different channel numbers to fit the input number of lifetime components. For the training, 60% of the cells were randomly selected as the training dataset, 10% of cells were defined as the validation dataset, and the remaining 30% were used to test the model (Supplementary Table S7). The network was trained using an NVIDIA GeForce RTX 2070 GPU, and the training parameters including learning rate, batch size, and epochs were tuned to achieve the best performance of the model. The learning rate was tested with values of 0.001, 0.0001, and 0.00001, while the batch size was evaluated for 8, 16, 32, 64, 128. All hyperparameter combinations were iteratively evaluated using 100 epochs to determine the optimal combination of learning rate and batch size that minimized error for the model’s prediction results. Finally, the performance of models with the best hyperparameters was monitored during training to determine the appropriate number of epochs. As a result, the learning rate was set as 0.0001, and the batch size was set at 16 with 100 epochs for training. The AUC, accuracy, precision, recall, precision-recall curve, and ROC curve of the prediction on the test dataset were used to evaluate the performance of CNN models. The prediction result was presented in a confusion matrix, where glycolysis inhibition cells were defined as the negative group, and the OXPHOS inhibition cells were defined as the positive group. The accuracy was calculated as the percentage of correctly classified data over the total number of the data. Precision was determined as the percentage of true positives out of all the positive predictions. The recall was calculated as the percentage of correctly predicted positive cases out of the total actual positive cases. To compare the performance of CNN with classical machine learning models, we trained three typical machine learning algorithms (random forest tree, support vector machine, and quadratic discriminant analysis) with the same datasets. We calculated the average value of non-zero pixels in each input image and used the average value as a feature to train the classical machine learning models for the classification of glycolysis-inhibited or OXPHOS-inhibited cells.

## RFT and SVM Hyperparameter Selection

An extensive examination of the number of trees was conducted by testing values across a range of scales (1, 5, 10, 20, 50, 100). We finally chose the number of trees to be 50, as it exhibited the least error in the 5-fold cross-validation (~8.47% for non-normalization, ~2.58% for normalization). Furthermore, polynomial and sigmoid kernels were compared for SVM to assess their impact on model performance. The linear kernel was selected as it had the best performance. As a result, the cross-validation results for different models demonstrated stable performance across different folds in predicting metabolic phenotypes with an accuracy of approximately 90%.

# Supplementary Figures and Tables

## Supplementary Figures


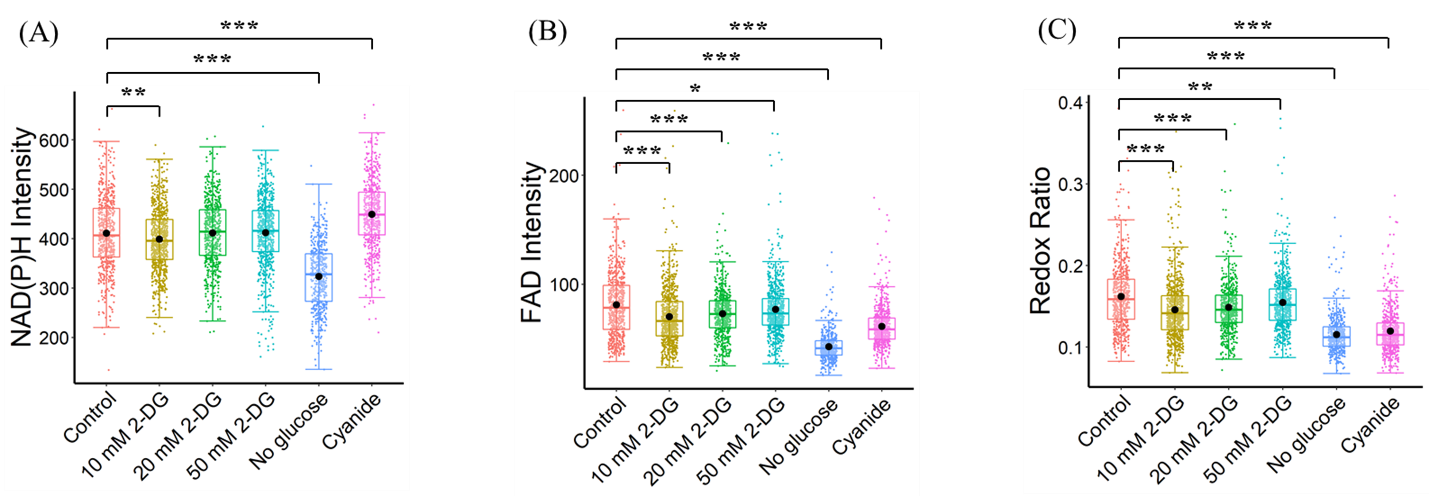


**Supplementary Figure 1.** Glycolysis and OXPHOS inhibition vary NAD(P)H and FAD intensity, and redox ratio. Comparison of (A) NAD(P)H intensity (B) FAD intensity (C) redox ratio (FAD/ (FAD + NAD(P)H)) of MCF7 cancer cells exposed to different metabolic environments. *P < 0.05, **P < 0.01, ***P < 0.001 for two-sided Wilcoxon test with Bonferroni correction for multiple comparisons. Substrates in each media: Control (25 mM glucose + 1 mM pyruvate), 2-DG (25 mM glucose + 1 mM pyruvate + 10/20/50 mM 2-DG), No glucose (50 mM pyruvate), Cyanide (25 mM glucose + 1 mM pyruvate + 4 mM NaCN).


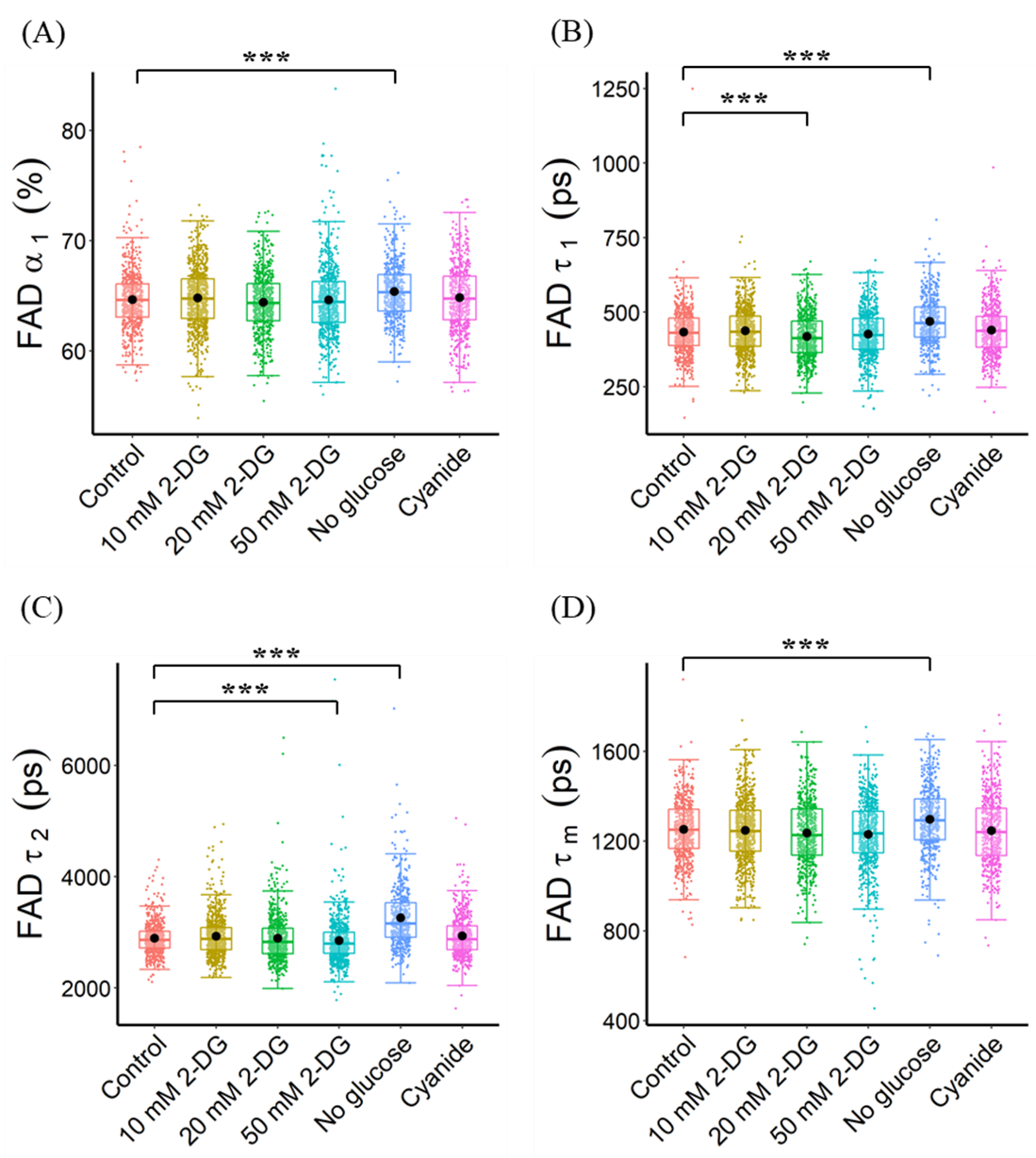


**Supplementary Figure 2.** Glycolysis and OXPHOS inhibition vary FAD lifetime. Comparison of (A) FAD *α_1_* (B) FAD *τ_1_* (C) FAD *τ_2_* (D) FAD *τ_m_* of MCF7 cancer cells exposed to different metabolic environments. ***P < 0.001 for two-sided Wilcoxon test with Bonferroni correction for multiple comparisons. Substrates in each media: Control (25 mM glucose + 1 mM pyruvate), 2-DG (25 mM glucose + 1 mM pyruvate + 10/20/50 mM 2-DG), No glucose (50 mM pyruvate), Cyanide (25 mM glucose + 1 mM pyruvate + 4 mM NaCN).


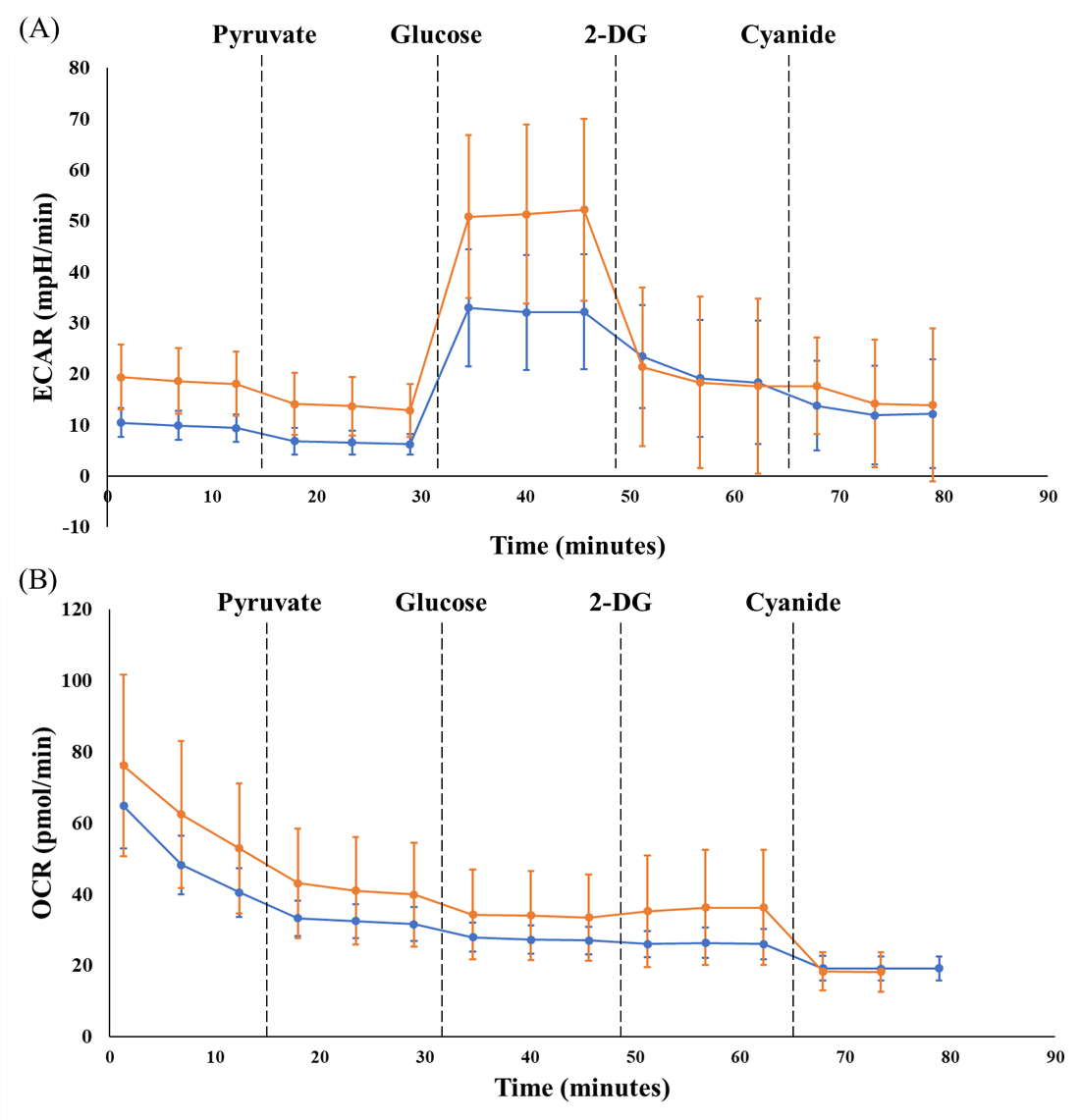


**Supplementary Figure 3.** Assay of mitochondrial respiration in MCF7 cells exposed to different metabolic environments. (A) Extracellular acidification rate (ECAR) and (B) oxygen consumption rate (OCR) were measured under basal conditions (no substrates) followed by the sequential addition of pyruvate (1 mM), glucose (100 mM), 2-DG (50 mM) as well as cyanide (4 mM), as indicated. Each color represents a cell group with a certain density. Orange corresponds to 10^6^ cells/ml, and blue corresponds to 5 x 10^5^ cells/ml.


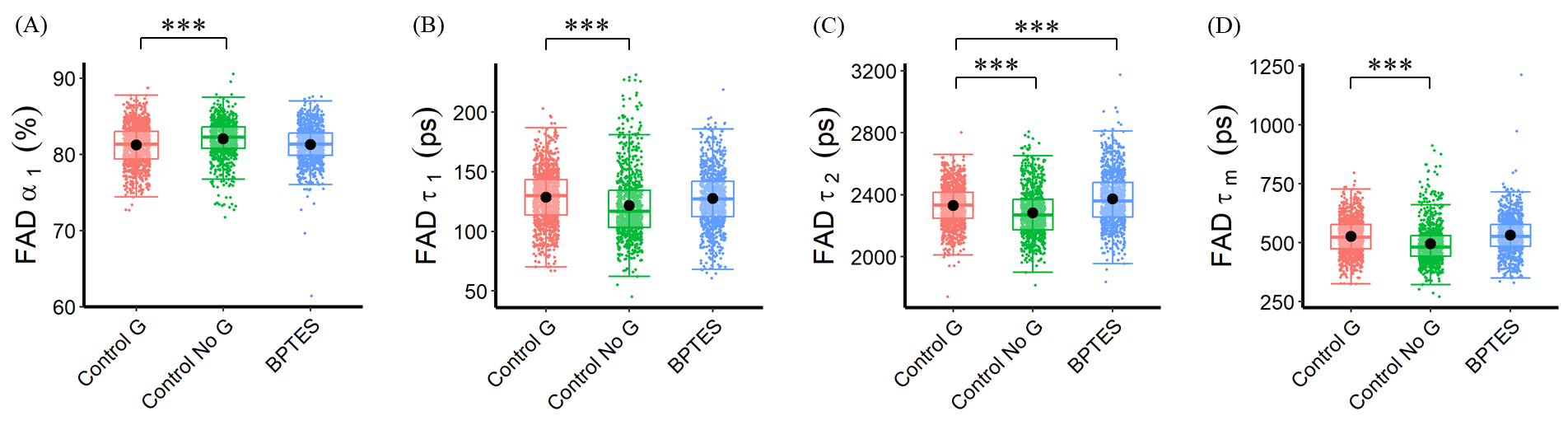


**Supplementary Figure 4.** Autofluorescence lifetime variations in response to glutaminolysis inhibition. (A) FAD *α_1_* (B) FAD *τ_1_* (C) FAD *τ_2_* (D) FAD *τ_m_* of MCF7 cells in response to glutaminolysis perturbations. ***P < 0.001 for two-sided Wilcoxon test with Bonferroni correction for multiple comparisons. Substrates in each media: Control G (25 mM glucose + 1 mM pyruvate + 2 mM glutamine), Control No G (25 mM glucose + 1 mM pyruvate), BPTES (25 mM glucose + 1 mM pyruvate + 2 mM glutamine + 10 µm BPTES).


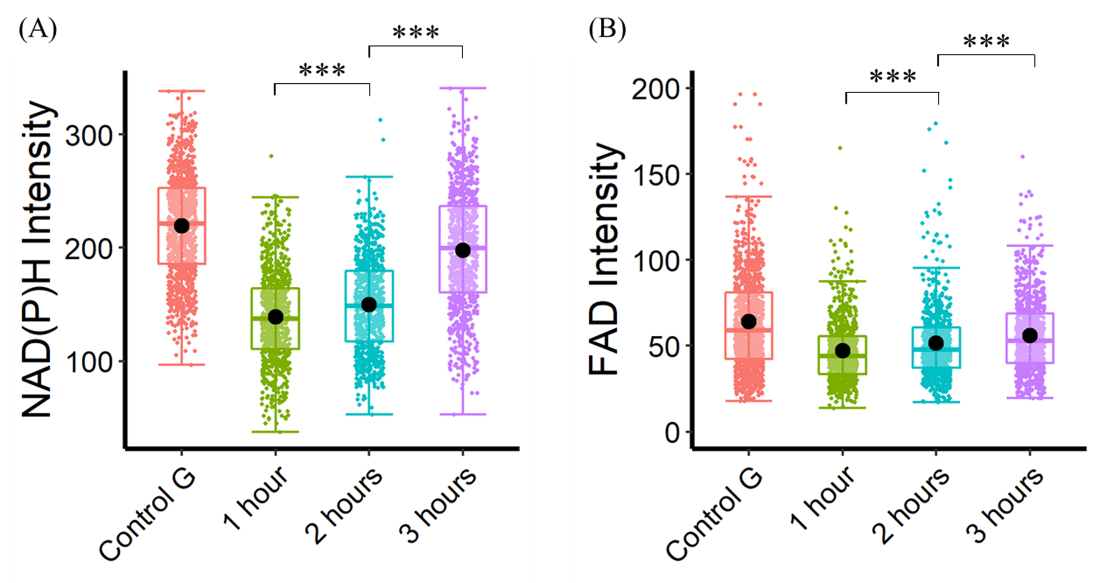


**Supplementary Figure 5.** Glutaminolysis effect on NAD(P)H and FAD intensity in MCF7 cancer cells. (A) NAD(P)H intensity (B) FAD intensity of cells exposed to only glutamine after 1 hour, 2 hours, and 3 hours. ***P < 0.001 for two-sided Wilcoxon test with Bonferroni correction for multiple comparisons. Substrates in each media: Control G (25 mM glucose + 1 mM pyruvate + 2 mM glutamine), 1 hour (no glucose + no pyruvate + 2 mM glutamine (1 hour)), 2 hours (no glucose + no pyruvate + 2 mM glutamine (2 hours)), 3 hours (no glucose + no pyruvate + 2 mM glutamine (3 hours)).


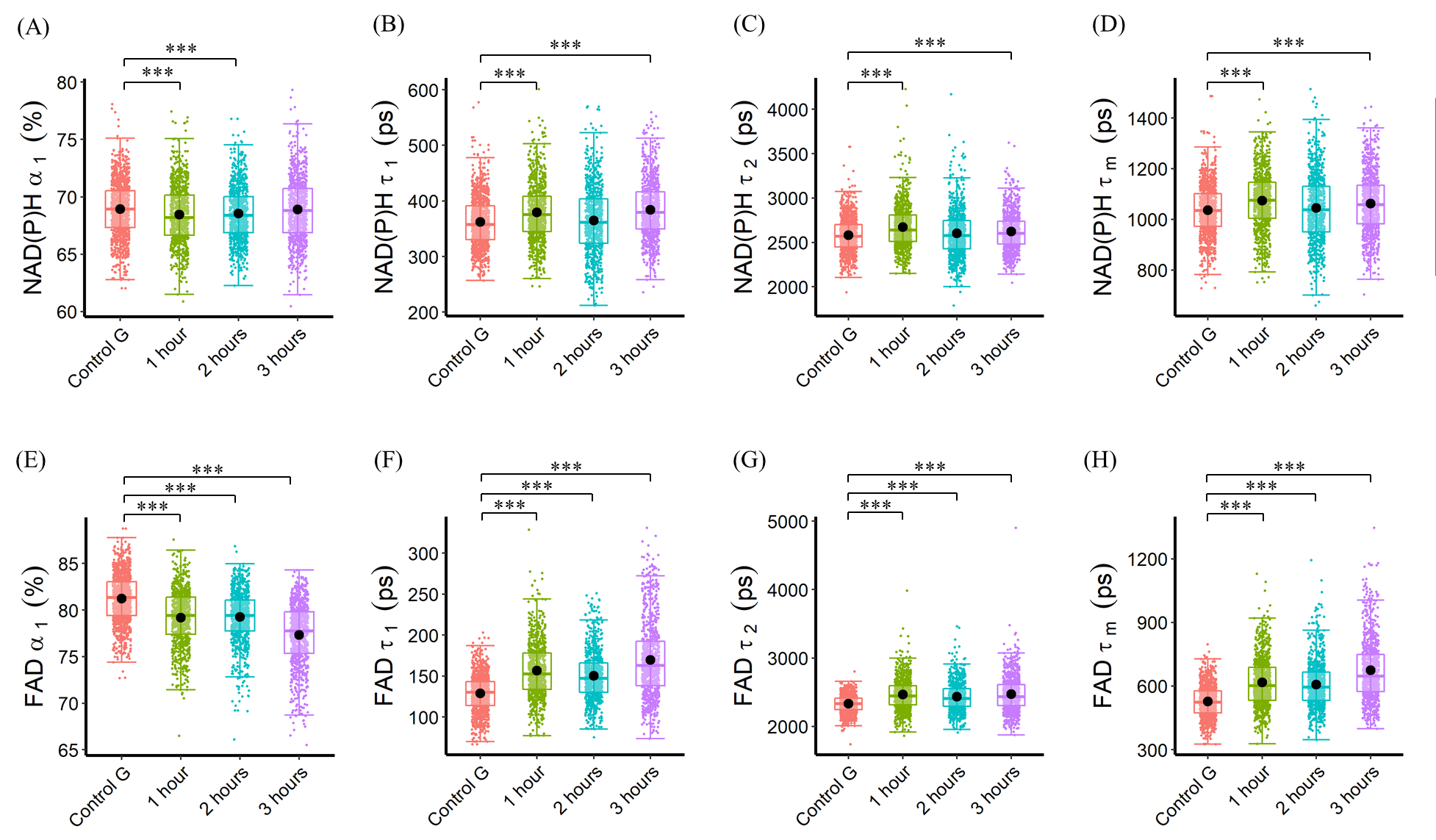


**Supplementary Figure 6.** Glutaminolysis effect on NAD(P)H and FAD fluorescence lifetime in MCF7 cancer cells over the time period. (A) NAD(P)H *α_1_* (B) NAD(P)H *τ_1_* (C) NAD(P)H *τ_2_* (D) NAD(P)H *τ_m_* (E) FAD *α_1_* (F) FAD *τ_1_* (G) FAD *τ_2_* (H) FAD *τ_m_* of cells exposed to only glutamine after 1 hour, 2 hours, and 3 hours. ***P < 0.001 for two-sided Wilcoxon test with Bonferroni correction for multiple comparisons. Substrates in each media: Control G (25 mM glucose + 1 mM pyruvate + 2 mM glutamine), 1 hour (no glucose + no pyruvate + 2 mM glutamine (1 hour)), 2 hours (no glucose + no pyruvate + 2 mM glutamine (2 hours)), 3 hours (no glucose + no pyruvate + 2 mM glutamine (3 hours)).


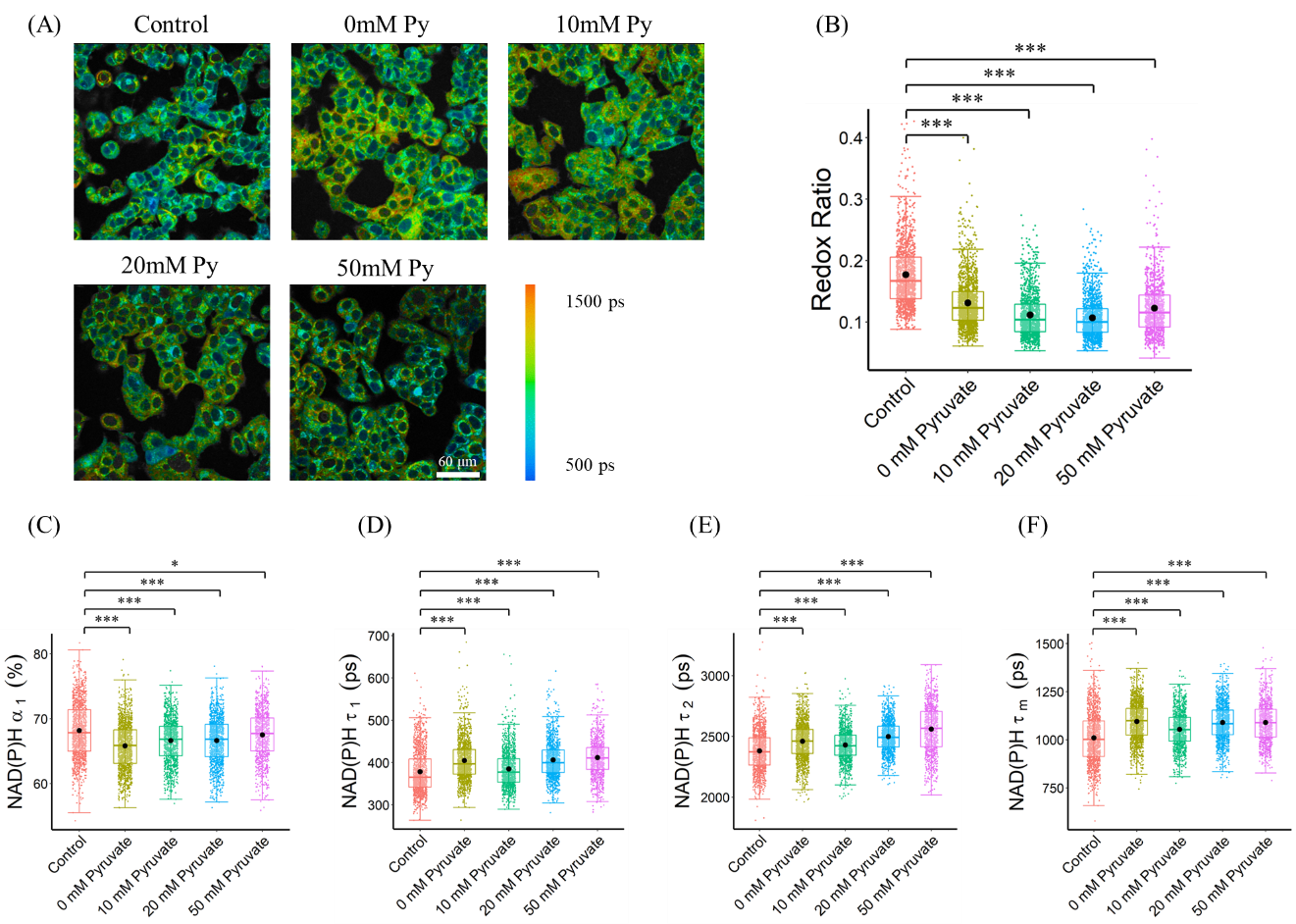


**Supplementary Figure 7.** NAD(P)H lifetime variations of cancer cells in different pyruvate assay groups. (A) Representative NAD(P)H *τ_m_* images of MCF7 cancer cells exposed to different pyruvate concentrations, Py, pyruvate; scale bar = 60 μm (B) Redox ratio (FAD/(FAD + NAD(P)H)) (C) NAD(P)H *α_1_* (D) NAD(P)H *τ_1_* (E) NAD(P)H *τ_2_* (F) NAD(P)H *τ_m_* of different pyruvate assay groups. *P < 0.05, ***P < 0.001 for two-sided Wilcoxon test with Bonferroni correction for multiple comparisons. Substrates in each media: Control (25 mM glucose + 1 mM pyruvate), Pyruvate (no glucose + no glutamine + 0/10/20/50 mM pyruvate).


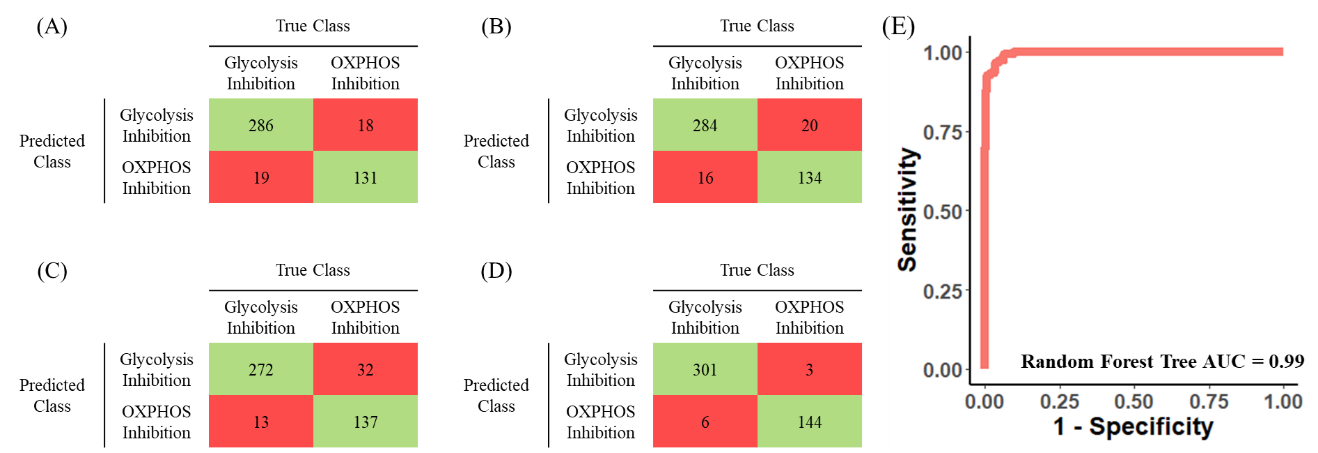


**Supplementary Figure 8.** Classification of cancer cell metabolic activities with autofluorescence lifetime features. Representative prediction result of (A) random forest tree (B) support vector machine (C) quadratic discriminant analysis models without feature normalization. (D) Representative prediction result of random forest tree with feature normalization by the corresponding control groups. (E) ROC curves of the test data for the random forest tree model with feature normalization by the corresponding control groups.


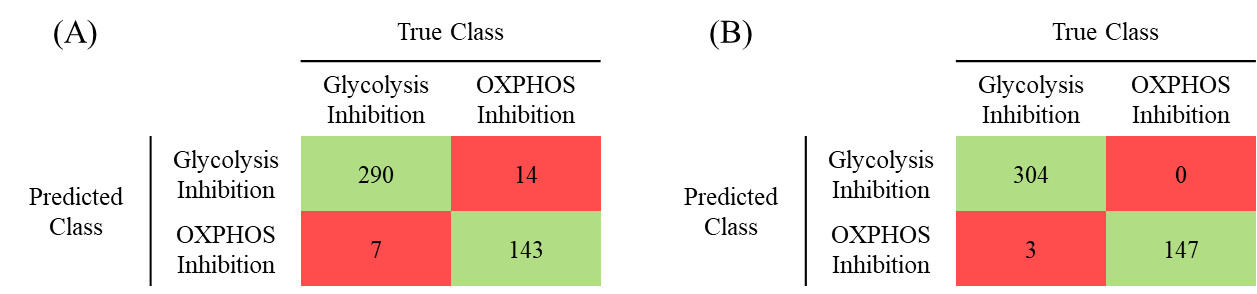


**Supplementary Figure 9.** Classification of MCF7 cell metabolic activities from autofluorescence lifetime features segmented from mitochondria or cytosol regions of each cell. Representative confusion matrix of random forest tree prediction results of models trained with lifetime features isolated from (A) cytosol (NAD(P)H-low intensity pixels), and (B) mitochondria (NAD(P)H-high intensity pixels).


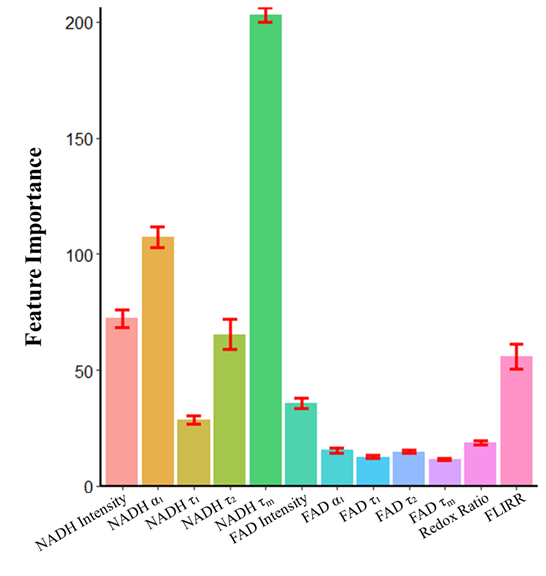


**Supplementary Figure 10.** Feature importance within the RFT model for classifying glycolytic versus oxidative cancer cells.


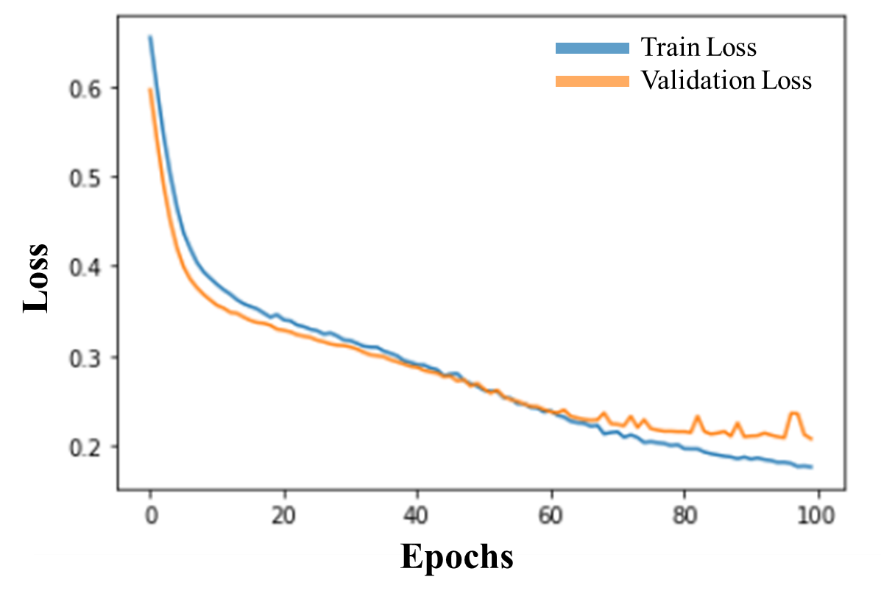


**Supplementary Figure 11.** Train and validation loss of CNN model (NAD(P)H *α₁* + *τ_1_* + *τ_2_* + *τ_m_* + Intensity) upon the number of training epochs. The loss function is the cross-entropy loss between true labels and predicted labels.


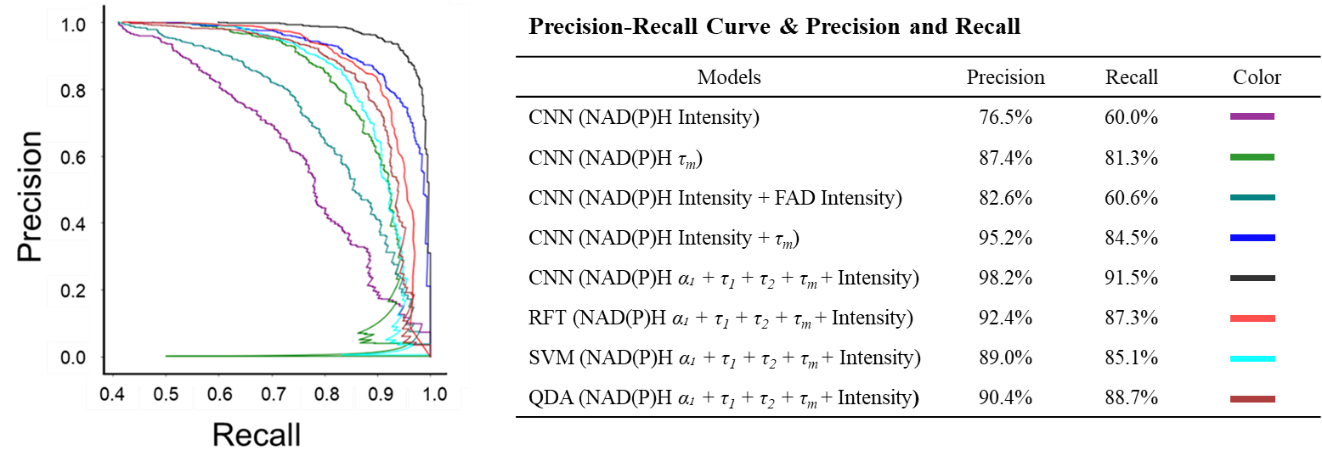


**Supplementary Figure 12.** Precision-recall curves, precision and recall for the test dataset (n = 1520) of each classifier built to predict metabolism as glycolysis inhibition or OXPHOS inhibition. RFT: random forest tree; SVM: support vector machine; QDA: quadratic discriminant analysis.


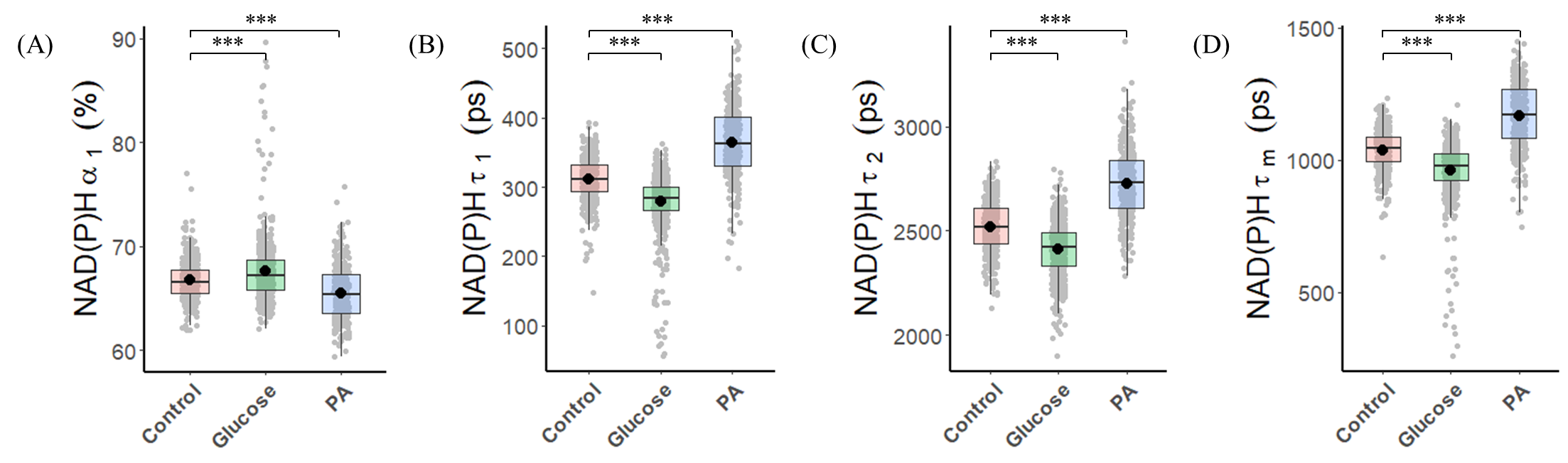


**Supplementary Figure 13.** Comparison of NAD(P)H lifetime components within HepG2 liver cancer cells in different metabolic environments. (A) NAD(P)H *α_1_* (B) NAD(P)H *τ_1_* (C) NAD(P)H *τ_2_* (D) NAD(P)H *τ_m_* of cells exposed to glucose and palmitate respectively. *P < 0.05, **P < 0.01, ***P < 0.001 for two-sided Wilcoxon test with Bonferroni correction for multiple comparisons.


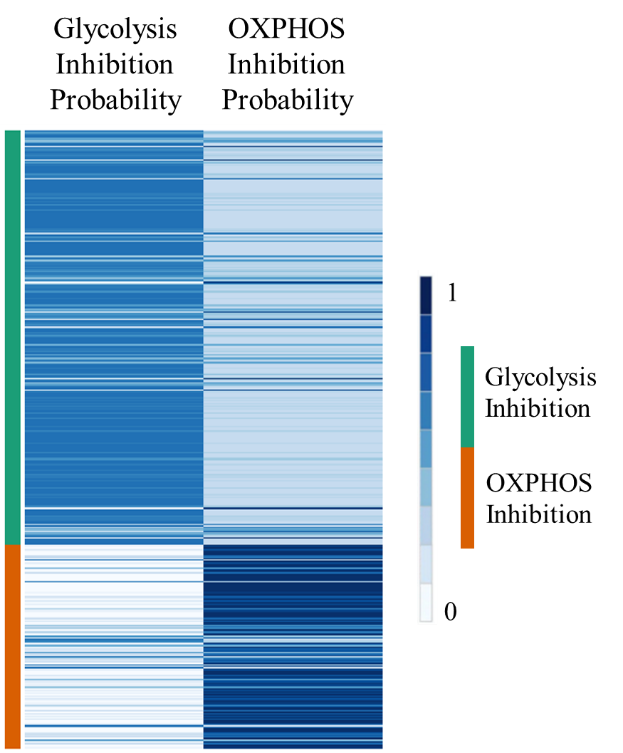


**Supplementary Figure 14.** Heatmap of prediction probability for test data (n = 454) to be classified as Glycolysis Inhibition or OXPHOS inhibition for the random forest tree machine learning model that predicts cellular metabolism from autofluorescence lifetime features. Each row represents a single cell, and the shade of blue indicates the probability of that cell having a specific metabolic phenotype as identified by the machine learning model.


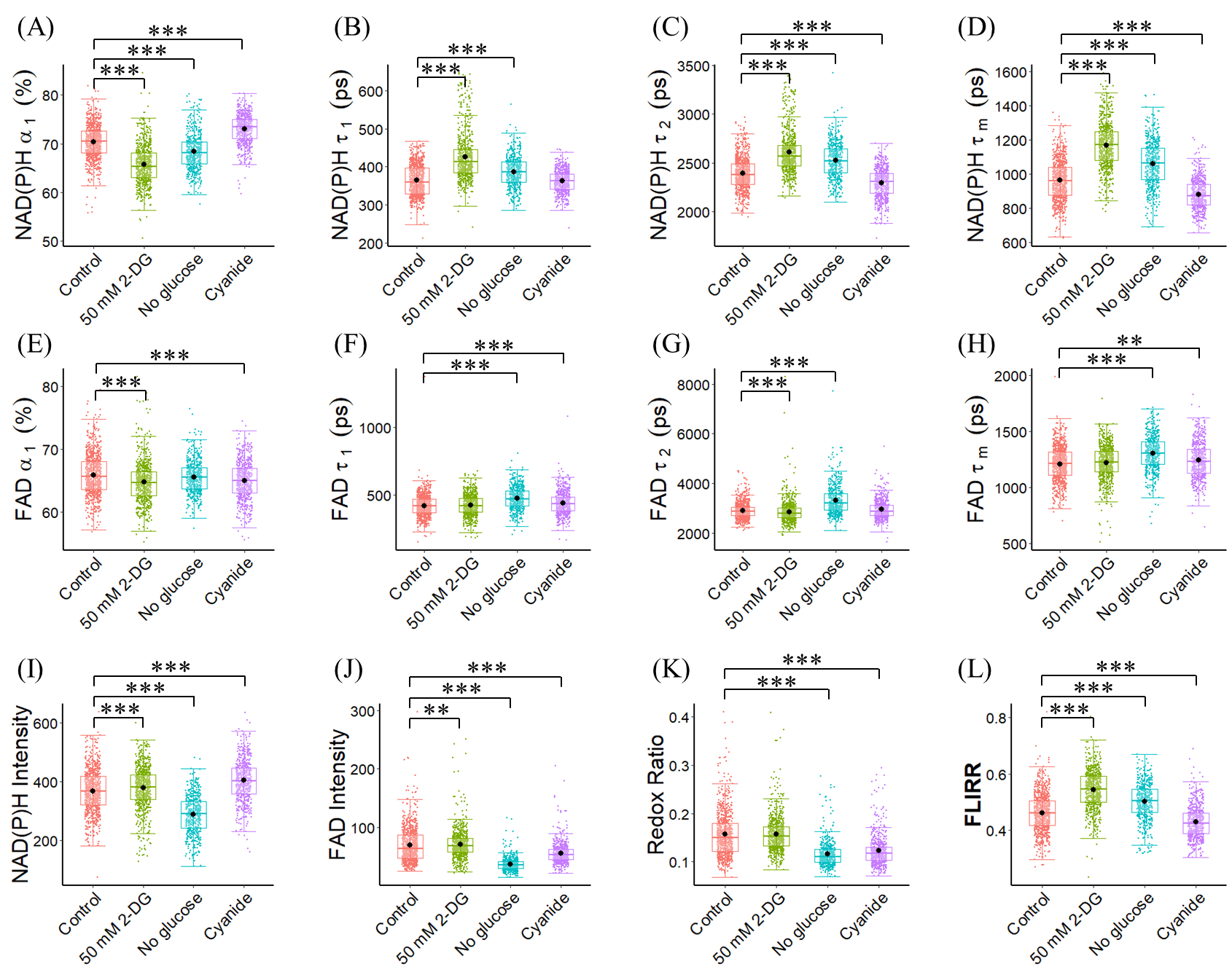


**Supplementary Figure 15.** Autofluorescence lifetime values of non-mitochondria compartments (low NAD(P)H intensity) of MCF7 cell cytosol for different metabolic phenotypes. Comparison of (A) NAD(P)H *α_1_* (B) NAD(P)H *τ_1_* (C) NAD(P)H *τ_2_* (D) NAD(P)H *τ_m_* (E) FAD *α_1_* (F) FAD *τ_1_* (G) FAD *τ_2_* (H) FAD *τ_m_* (I) NAD(P)H intensity (J) FAD intensity (K) redox ratio (FAD/ (FAD + NAD(P)H)) (L) FLIRR of MCF7 cells exposed to different metabolic environments. ***P < 0.001 for two-sided Wilcoxon test with Bonferroni correction for multiple comparisons. Substrates in each media: Control (25 mM glucose + 1 mM pyruvate), 2-DG (25 mM glucose + 1 mM pyruvate + 50 mM 2-DG), No glucose (50 mM pyruvate), Cyanide (25 mM glucose + 1 mM pyruvate + 4 mM NaCN).


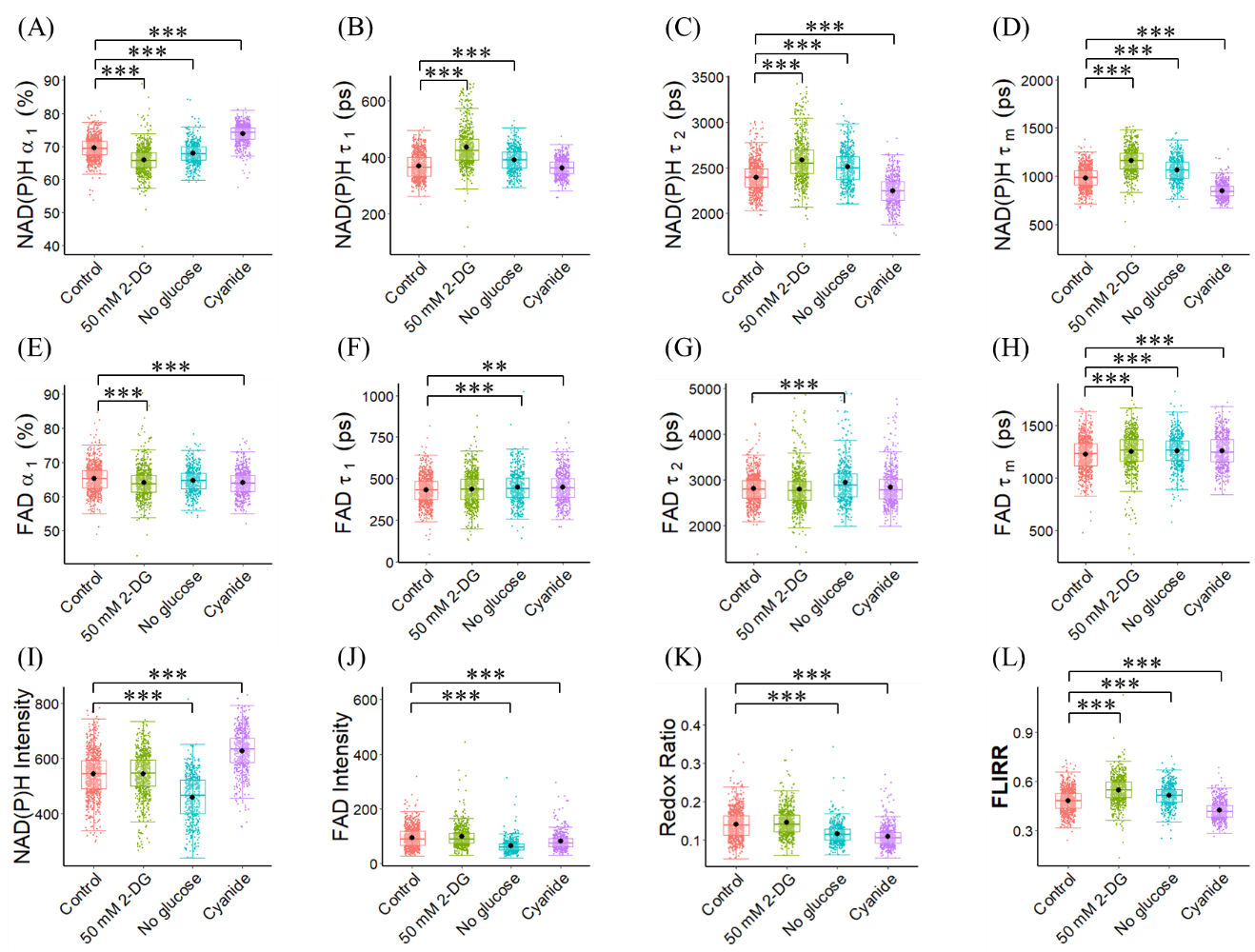


**Supplementary Figure 16.** Autofluorescence lifetime values in mitochondria (high-NAD(P)H intensity pixels) of MCF7 cells for different metabolic phenotypes. Comparison of (A) NAD(P)H *α_1_* (B) NAD(P)H *τ_1_* (C) NAD(P)H *τ_2_* (D) NAD(P)H *τ_m_* (E) FAD *α_1_* (F) FAD *τ_1_* (G) FAD *τ_2_* (H) FAD *τ_m_* (I) NAD(P)H intensity (J) FAD intensity (K) redox ratio (FAD/ (FAD + NAD(P)H)) (L) FLIRR of cancer cells exposed to different metabolic environments. ***P < 0.001 for two-sided Wilcoxon test with Bonferroni correction for multiple comparisons. Substrates in each media: Control (25 mM glucose + 1 mM pyruvate), 2-DG (25 mM glucose + 1 mM pyruvate + 50 mM 2-DG), No glucose (50 mM pyruvate), Cyanide (25 mM glucose + 1 mM pyruvate + 4 mM NaCN).


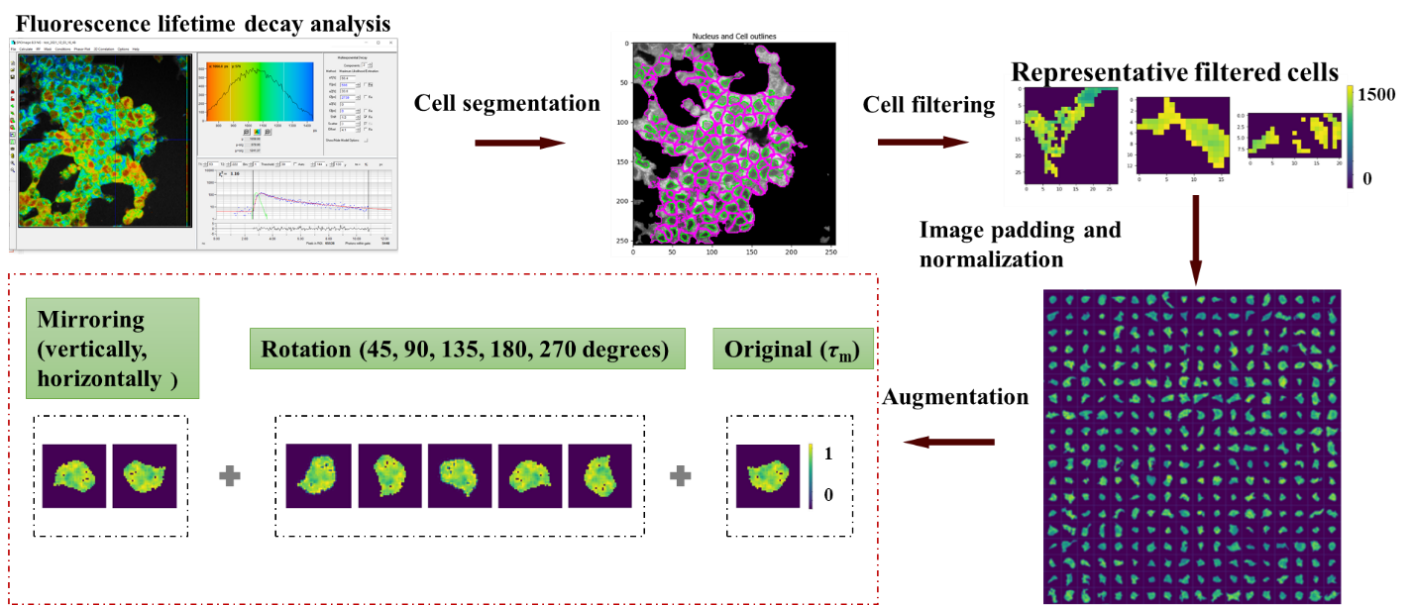


**Supplementary Figure 17.** Cancer cell image data preprocessing workflow. Fluorescence lifetime decay was analyzed by SPCImage to extract different endpoints (intensity, *α_1_*, *τ_1_*, *τ_2_*), and the cells were then segmented by CellProfiler from NAD(P)H intensity images. The images were padded to be 40 x 40, and augmented by rotating different angles (45, 90, 135, 180, 270) and mirroring.

## Supplementary Tables

**Table S1.** Changes of NAD(P)H and FAD fluorescence lifetime under different metabolic perturbations

|  | **NAD(P)H** | | | | | **FAD** | | | | | **Redox Ratio** | |
| --- | --- | --- | --- | --- | --- | --- | --- | --- | --- | --- | --- | --- |
|  | *α_1_* | *τ_1_* | *τ_2_* | *τ_m_* | *Int* | *α_1_* | *τ_1_* | *τ_2_* | *τ_m_* | *Int* | *IRR* | *FLIRR* |
| 10 mM 2-DG | ↓ | ↔ | ↑ | ↑ | ↓ | ↔ | ↔ | ↔ | ↔ | ↓ | ↓ | ↑ |
| 20 mM 2-DG | ↓ | ↑ | ↑ | ↑ | ↔ | ↔ | ↓ | ↔ | ↔ | ↓ | ↓ | ↑ |
| 50 mM 2-DG | ↓ | ↑ | ↑ | ↑ | ↔ | ↔ | ↔ | ↓ | ↔ | ↓ | ↓ | ↑ |
| No Glucose | ↓ | ↑ | ↑ | ↑ | ↓ | ↑ | ↑ | ↑ | ↑ | ↓ | ↓ | ↑ |
| Cyanide | ↑ | ↓ | ↓ | ↓ | ↑ | ↔ | ↔ | ↔ | ↔ | ↓ | ↓ | ↓ |

Int, Intensity; IRR, intensity redox ratio (FAD/ (FAD +NAD(P)H)), FLIRR, fluorescence lifetime redox ratio (NAD(P)H *α_2_*/ FAD *α_1_*). ↑, increase; ↓, decrease; ↔, no change.

**Table S2.** Prediction results from the normalized random forest tree model for MCF7 cell metabolism for the 10 mM 2-DG, 20 mM 2-DG, 0 mM pyruvate, 10 mM pyruvate, 20 mM pyruvate, and 50 mM pyruvate groups. 2-DG inhibits glycolysis. Pyruvate stimulates OXPHOS, Glutamine stimulates glutaminolysis.

|  | 10 mM 2-DG | 20 mM 2-DG | 10 mM Pyruvate | 20 mM Pyruvate | 50 mM Pyruvate | Glutamine 1 hour | Glutamine 2 hours | Glutamine 3 hours |
| --- | --- | --- | --- | --- | --- | --- | --- | --- |
| Glycolysis Inhibition | 82.6% (659) | 85.9% (531) | 98.5% (1152) | 98.9% (1217) | 99.2% (924) | 99.3% (812) | 96.7% (765) | 97.1% (857) |
| OXPHOS Inhibition | 17.4% (139) | 14.1% (87) | 1.5%  (17) | 1.1%  (13) | 0.8%  (7) | 0.7% (6) | 3.3% (27) | 2.9% (86) |

**Table S3.** Prediction result of T cells with CNN model

|  | Glycolysis Inhibition | OXPHOS Inhibition |
| --- | --- | --- |
| Activated T cells | 1 (0.4%) | 262 (99.6%) |
| Quiescent T cells | 5 (1.7%) | 285 (98.3%) |

**Table S4.** The concentration of metabolic substrates in each group

| Media | The concentration of metabolic substrates |
| --- | --- |
| Control Media 1 (No glutamine) | glucose (25 mM), pyruvate (1 mM), no glutamine |
| 10 mM 2-DG | glucose (25 mM), pyruvate (1 mM), no glutamine, 2-DG (10 mM, 1 hour) |
| 20 mM 2-DG | glucose (25 mM), pyruvate (1 mM), no glutamine, 2-DG (20 mM, 1 hour) |
| 50 mM 2-DG | glucose (25mM), pyruvate (1 mM), no glutamine, 2-DG (50 mM, 1 hour) |
| No glucose | no glucose, pyruvate (50 mM, 1 hour), no glutamine |
| Cyanide | glucose (25mM), pyruvate (1 mM), no glutamine, NaCN (4 mM) |
| Control Media 2 (0 mM pyruvate) | no glucose, no pyruvate, no glutamine |
| 10 mM pyruvate | no glucose, pyruvate (10 mM, 1 hour), no glutamine |
| 20 mM pyruvate | no glucose, pyruvate (20 mM, 1 hour), no glutamine |
| 50 mM pyruvate | no glucose, pyruvate (50 mM, 1 hour), no glutamine |
| Control Media 3 | glucose (25mM), pyruvate (1 mM), glutamine (2 mM) |
| BPTES | glucose (25 mM), pyruvate (1 mM), glutamine (2 mM), BPTES (10 µm, 1 hour) |
| Glutamine 1 | no glucose, no pyruvate, glutamine (2 mM, 1 hour) |
| Glutamine 2 | no glucose, no pyruvate, glutamine (2 mM, 2 hours) |
| Glutamine 3 | no glucose, no pyruvate, glutamine (2 mM, 3 hours) |

**Table S5.** Number of Cells in each group

| Target Metabolic Pathways | Metabolic Manipulation | Treatments | Image Number | Experimental Replicates | Cell Number |
| --- | --- | --- | --- | --- | --- |
| Glycolysis & OXPHOS | Control | CM1 | 16 | 3 | 644 |
|  | OXPHOS Maximum | 10 mM 2-DG | 14 | 3 | 798 |
|  |  | 20 mM 2-DG | 16 | 3 | 618 |
|  |  | 50 mM 2-DG | 17 | 3 | 707 |
|  |  | No glucose* | 16 | 3 | 511 |
|  | Glycolysis Maximum | Cyanide | 17 | 3 | 601 |
| Pyruvate to acetyl-CoA | Control | CM1 | 20 | 3 | 1289 |
|  | Pyruvate Concentration | 0 mM pyruvate | 19 | 3 | 1481 |
|  |  | 10mM pyruvate | 19 | 3 | 1169 |
|  |  | 20mM pyruvate | 19 | 3 | 1230 |
|  |  | 50mM pyruvate | 16 | 3 | 931 |
| Glutaminolyisis | Control | CM3 | 17 | 3 | 1113 |
|  | Glutaminolysis  Inhibition | BPTES | 15 | 3 | 919 |
|  |  | No glutamine | 14 | 3 | 879 |
|  | Glutaminolysis  Compensation | Glutamine 1^†^ | 15 | 3 | 830 |
|  |  | Glutamine 2^‡^ | 14 | 3 | 803 |
|  |  | Glutamine 3^§^ | 14 | 3 | 894 |

*No glucose: CM2 with 50mM pyruvate; ^†^Glutamine 1, DMEM media only with glutamine for 1 hour; ^‡^Glutamine 2, DMEM media only with glutamine for 2 hours; ^§^Glutamine 3, DMEM media only with glutamine for 3 hours.

**Table S6.** Number of liver cancer cells and T cells

| Liver cancer cells | Cell Number | T cells | Cell Number |
| --- | --- | --- | --- |
| Control | 451 | Activated T cells | 263 |
| Glucose | 596 | Quiescent T cells | 290 |
| Palmitate | 334 |  |  |

**Table S7.** Number of cells for CNN model development and testing

|  | Original | Training (60%) | Testing (30%) | Validation (10%) | Augmentation |
| --- | --- | --- | --- | --- | --- |
| Glycolysis inhibition | 2985 | 1196 | 897 | 299 | 14312 |
| OXPHOS inhibition | 2082 | 1251 | 623 | 208 | 10008 |
| Sum | 5067 | 3040 | 1520 | 507 | 24320 |

## References

Drozdowicz-Tomsia, K., Anwer, A.G., Cahill, M.A., Madlum, K.N., Maki, A.M., Baker, M.S., and Goldys, E.M. (2014). Multiphoton fluorescence lifetime imaging microscopy reveals free-to-bound NADH ratio changes associated with metabolic inhibition. *J Biomed Opt* 19**,** 086016.

Walsh, A.J., Cook, R.S., Manning, H.C., Hicks, D.J., Lafontant, A., Arteaga, C.L., and Skala, M.C. (2013). Optical metabolic imaging identifies glycolytic levels, subtypes, and early-treatment response in breast cancer. *Cancer Res* 73**,** 6164-6174.

Walsh, A.J., Mueller, K.P., Tweed, K., Jones, I., Walsh, C.M., Piscopo, N.J., Niemi, N.M., Pagliarini, D.J., Saha, K., and Skala, M.C. (2021). Classification of T-cell activation via autofluorescence lifetime imaging. *Nat Biomed Eng* 5**,** 77-88.

Y Lecun, B.B., Js Denker, D Henderson, Re Howard, W Hubbard, Ld Jackel (1990). "Handwritten digit recognition with a back-propagation network", in: *Advances in neural information processing systems 2, NIPS 1989.* Morgan Kaufmann Publishers).
